# Molecular resilience: genetic analysis of multiple-stress tolerance (osmotic, salinity, cold and heat) during potato (*Solanum tuberosum* L.) microtuberization

**DOI:** 10.3389/fpls.2026.1828205

**Published:** 2026-05-29

**Authors:** Lisset Herrera-Isidron, Ilse Araceli Careaga-Rojas, Braulio Uribe-Lopez, Andrea-Maria Navarro-Vega, Aaron Barraza, Eliana Valencia-Lozano, José Luis Cabrera-Ponce

**Affiliations:** 1 Unidad Profesional Interdisciplinaria de Ingeniería Campus Guanajuato (UPIIG), Instituto Politécnico Nacional, Silao de la Victoria, Guanajuato, Mexico; 2 Departamento de Biotecnologia y Bioquimica, Centro de Investigación y de Estudios Avanzados del Instituto Politécnico Nacional (IPN), Irapuato, Guanajuato, Mexico; 3 Laboratorio Biotechnolgika, La Paz, Baja California Sur, Mexico; 4 Laboratorio de Investigación Interdisciplinaria (LII), Universidad Nacional Autónoma de México, Escuela Nacional de Estudios Superiores, León de los Aldama, Guanajuato, Mexico; 5 Laboratorio Nacional PlanTECC, Departamento de Ingeniería Genética, Centro de Investigación y de Estudios Avanzados del Instituto Politécnico Nacional (IPN), Irapuato, Guanajuato, Mexico

**Keywords:** cold, drought, H_2_S, heat, microtuberization, multiple-stress, potato, salinity

## Abstract

**Introduction:**

Multiple-stress is defined as the simultaneous or sequential exposure of plants to multiple abiotic constraints, which triggers regulatory programs that differ fundamentally from single-stress responses. In potato (*Solanum tuberosum* L.), drought, salinity, heat, and cold severely impair tuber development, yet the molecular architecture underlying resilience to combined stress remains unclear. We hypothesized that multi-stress conditions activate an integrated regulatory network linking tuber induction with stress-responsive metabolic and redox pathways.

**Methods:**

RNA-seq profiling of microtuberization under combined osmotic, salinity, heat, and cold stress was performed. Differential expression analysis identified shared differentially expressed genes (DEGs). A subset of upregulated genes was used for protein–protein interaction (PPI) network construction. Comparative regulatory analyses were performed, and selected genes were validated by qPCR. Statistical analyses were conducted to assess differential expression and network enrichment.

**Results:**

A total of 2,046 shared DEGs were identified, including 1,212 upregulated and 834 downregulated genes. A PPI network constructed from 1,475 unique upregulated genes revealed 317 highly interconnected components. Network analysis identified the StSP6A–FD tuberigen complex as a central regulatory hub integrating developmental signaling with phenylpropanoid metabolism, oxylipin biosynthesis, and redox regulation. Multiple components were associated with hydrogen sulfide (H₂S) signaling, suggesting redox–gasotransmitter integration.

**Discussion:**

Comparative regulatory analysis revealed conservation of the ERF–NAC–MYB–bZIP transcription factor framework, along with expansion of stress-responsive modules. Collectively, these findings establish a mechanistic framework linking tuber induction with adaptive metabolic remodeling under multi-stress conditions.

## 1 Introduction

Potato, *Solanum tuberosum* L., is the fourth most important food crop worldwide and a major contributor to global food security (Eed et al., 2025; FAOSTAT, 2026). It sustains more than a billion people, with an average per capita consumption of 33.3 kg during the period 2017-2021 (Varga and Đurović, 2023). Consequently, yield instability can translate into socioeconomic vulnerability and increased food insecurity.

Potato originated approximately nine million years ago in South America (Miocene Epoch; Neogene Period, Cenozoic Era) (Messeder et al., 2024), through interspecific hybridization between a wild tomato-like ancestor and a tuber-bearing member of the Etuberosum group in the Andes (Zhang et al., 2025).

During its early evolution, potatoes produced highly resilient tubers under conditions of volcanic activity, declining atmospheric CO_2,_ and cooler, drier climates, factors that favored adaptation to fluctuating environments. The domesticated potato *S. tuberosum* L., which was derived from a toxic wild tuber to become a nutritional staple, was driven by both cultural innovations, such as chuño, a freeze-drying technique that reduced glycoalkaloids like α-solanine, and natural selection under extreme highland conditions (Inquilla-Mamani and Chambi-Apaza, 2019). This process involved hybridization among wild species (*S. candolleanum*, *S. chacoense*, *S. brevicaule*), followed by spontaneous polyploidization (Hardigan et al., 2017).

This genetic foundation enabled the emergence of phenotypes adapted to drought, frost, and high-altitude environments. Its domestication and global dissemination to Europe enabled wide cultivation but also introduced new biotic pressures such as the late blight caused by *Phytophthora infestans*, which led to the Irish Potato Famine and spurred breeding efforts increasingly reliant on introgressed resistance genes from wild type relatives.

Climate change exacerbates these challenges by increasing temperatures, drought frequency, erratic precipitation, and soil salinization. Projected warming is expected to reduce potato productivity by 10–19% by 2039 and 18–32% by the 2050s (Hijmans, 2003).

As a cool temperate adapted species, potato is particularly susceptible to drought and heat (Monneveux et al., 2013; Qin et al., 2022; Levy and Veilleux, 2007; Hamoud et al., 2025), salinity (Chourasia et al., 2021), and cold (Li et al., 2025). In production environments, these multiple stresses (MST) often co-occur and can be more damaging than individual stresses (Zhao et al., 2025; George et al., 2017).

Combined-stress responses have been examined using integrative multi-omics together with network-inference (e.g. co-expression or curated knowledge networks) (Rasmussen et al., 2013; Demirel et al., 2020; Tan et al., 2023; Zagorščak et al., 2025). These frameworks enable the identification of responses specific to stress combination, which are often not predictable from single-stress profiles, and organize them into functionally coherent modules while highlighting hub regulators that coordinate cross-stress crosstalk.

Transcriptomic studies have revealed stress-responsive networks in potato, identifying transcription factors (TFs), hormonal regulators, and metabolic shifts involved in adaptation to abiotic and biotic stresses (George et al., 2017) (Valencia-Lozano et al., 2022, 2023), highlighted co-expression networks and ancestral gene modules involved in tuberization and environmental adaptation, while our previous work identified key genes regulating microtubers (MTs) development under combined osmotic, salinity, cold, and heat stresses, defining essential MST tolerance modules (Herrera-Isidron et al., 2024).

Tuberization is tightly regulated by environmental and hormonal cues (Aksoy et al., 2021; Dutt et al., 2017; Wang et al., 2018; George et al., 2017). Disruptions during tuber initiation or bulking directly reduce yield and quality, affecting both the food supply and propagation material (Zierer et al., 2021). Accordingly, efforts to improve stress resilience have increasingly shifted from single-gene targets toward pathway- and network-level regulators, given the limited and context-dependent success of single-gene engineering under abiotic stress.

In the present study, we extend our previous work by providing a deeper systems-level analysis of potato MTs development under combined abiotic stress. We integrated RNA-seq with STRING-based network inference and qPCR validation to expand the regulatory architecture linking developmental control with redox homeostasis, as well as cell wall and membrane remodeling (Szklarczyk et al., 2025).

## 2 Materials and methods

### 2.1 Plant material

Potato *S. tuberosum* cv. Alpha plantlets were propagated through shoot proliferation under *in vitro* conditions in MS medium (Murashige and Skoog, 1962), supplemented with 10 g/L sucrose (CAT 57-50–1 Sigma Aldrich, St. Louis, MO, USA), 3 g/L activated charcoal (CAT: 242276 Sigma-Aldrich, St. Louis, MO, USA), and solidified with 3 g/L gelrite (GELZAN CAT. G1910 Sigma Aldrich, St. Louis, MO, USA) at pH 5.8. Shoots were incubated at 25/17 °C under fluorescent light at 25 µmol/m^2^/s of irradiance. Stolon explants harvested from propagated shoots were subsequently used for MTs development.

MTs development was followed as indicated by Herrera-Isidron et al., 2021. Stolon explants were cultured in MR8-G6-2iP (8% sucrose, 6 g/L gelrite, 3 g/L activated charcoal, 10 mg/L 2iP, pH 5.8) and incubated in darkness at 25 °C/17 °C for 15 days (Control). This represents a baseline osmotic microtuberization condition rather than a stress-free control.

The MST treatments consisted of a sequential application of stress factors, rather than simultaneous exposure, and were designed to reproduce a defined stress regime during microtuberization rather than to isolate individual stress effects, according to Herrera-Isidron et al. (2024). Importantly, the MST represents a perturbation of the microtuberization baseline through this sequential stress exposure. Stolon explants were cultured in MR8-G6-2iP with NaCl 50 mM (osmotic/salinity stress), followed by heat (38 °C) for 24 h (osmotic-salinity-heat stress) and then by cold (4 °C) for 24 h (osmotic-salinity-heat-cold stress). Stolon explants completed the remaining 15 days of incubation at a 25 °C/17 °C cycle.

The viability of MTs development under both control and MST conditions was evaluated through plant regeneration assays. These were conducted using five biological replicates per condition. MTs were cultivated in MS medium supplemented with sucrose 30 g/L, activated charcoal 3 g/L, gelrite 3 g/L. Regenerated plants were established in soil conditions until maturity under controlled temperature and light conditions. Statistical analysis using ANOVA was conducted.

To evaluate the effect of H_2_S signaling in MTs development, the MST treatment was applied as described above, supplemented with two concentrations of the H_2_S precursor; 10 µM and 1 mM of NaHS (Sodium hydrosulfide flakes; CAS 207683, Kandelium México). Five biological replicates per treatment were used, with three independent experimental repeats. Explants were incubated for fifteen days under the corresponding treatment conditions.

Following the incubation period, MT development was assessed by quantifying the surface area of the MTs. Images of the MTs were captured, and the MT area was measured using ImageJ software, and area values were normalized prior to statistical analysis.

#### 2.1.1 RNA extraction and library preparation for transcriptome sequencing

Total RNA isolation was performed using Trizol reagent (Invitrogen, Carlsbad, CA, USA). The RNA concentration was quantified spectrophotometrically at 260 nm, and purity was verified by assessing the 260 nm/280 nm absorbance ratio. RNA integrity was confirmed via electrophoresis on a 1% (w/v) agarose gel.

Library preparation and transcriptome sequencing were performed by GENEWIZ, Plainfield, NJ, USA. Illumina HiSeq 2500 (Illumina, San Diego, CA, USA) was used for sequencing. Sequenced reads were tested for quality using the FastQC software package (http://www.bioinformatics.babraham.ac.uk/projects/fastqc/) and preprocessed to remove sequence adapters and low-quality bases using the software Trimmomatic (Bolger et al., 2014).

RNAseq reads were aligned to the *S. tuberosum* reference genome available in Phytozome v12.1. (https://phytozome.jgi.doe.gov/pz/portal.html) with the STAR aligner v.2.5.2b (Dobin et al., 2013). In this step, the BAM (Binary Alignment/Map) files were generated. Subsequently, a count and set of transcripts were made using the featureCounts program of the Subread v.1.5.2 package (Liao et al., 2014).

#### 2.1.2 Quantification of gene expression levels and differential expression analysis

Gene expression levels were estimated by FPKM (Fragments Per Kilobase of Transcript Per Million mapped reads) values. Differential expression between MR1-MST and MR8-MST was analyzed with DESeq2 v1.12.4, applying the Benjamini–Hochberg procedure to control the false discovery rate.

Genes with an adjusted p-value < 0.05 and fold change > 1.5 (log2) were considered differentially expressed. Functional annotation and ontology analysis were performed using Blast2GO, classifying genes into biological processes, molecular functions, and cellular components. This approach allowed the identification of stress-responsive pathways and regulatory networks involved in potato MTs development under MST conditions.

#### 2.1.3 Protein-protein-Interaction analysis

Protein–protein interaction (PPI) networks contribute to the framework for many biological pathways, making PPI prediction essential to understanding protein function and regulatory mechanisms. Differentially expressed genes (DEGs) were BLASTed (blastx) against the genome of a related species using the STRING-database v12.0 with a confidence score of 0.500 (Szklarczyk et al., 2025).

The network was built by retrieving homologous genes in the *S. tuberosum* genome from the Sol Genomics Network, with identifiers cross-referenced using UniProt (UniProt, 2021) and NCBI Gene (Sherry et al., 2001). For functional inference, potato proteins exhibiting ≥ 60% sequence identity with *A. thaliana* orthologs were selected.

The 60% cutoff was adopted to ensure the selection of interologs and to provide a well-supported foundation for functional orthology. According to Rost (1999), sequence alignments unequivocally distinguish between protein pairs with similar and non-similar structures when sequence identity is high (>40% for long alignments). By exceeding it, we distance our analysis from the “twilight zone” (20-35% identity), where structural divergence significantly increases the risk of false-positive interactions due to limited homology.

#### 2.1.4 Transcription factor analysis

To decipher the regulatory architecture governing the observed transcriptional response, we subjected the core PPI-network of 1475 upregulated genes to a targeted Transcription Factor (TF) profiling. Potato-specific TFs were characterized through protein functional annotation, while their PPI-network was computationally constructed via orthology-based inference with *A. thaliana*.

This was further refined through Principal Component Analysis (PCA) to capture the primary dimensions of variance within the dataset.

#### 2.1.5 Validation of transcriptome data by qPCR in MST response

RNA isolation and cDNA synthesis were carried out, and subsequent amplification was performed using SYBR™ Green Master Mix (ThermoFisher, Waltham, MA, USA) in a Real-Time PCR System (CFX96 BioRad, Hercules, CA, USA).

Ten genes were chosen to validate the transcriptome analysis of potato MT development under MST conditions. *PER7* XM_006364783.2 (*M1B2E4*), peroxidase 7; *PAL1* XM_006367472.2 (*M1D3Y2*), phenylalanine ammonia-lyase 1; *LOX1* NM_001287987.1 (*M1AQS2*), linoleate 9S-lipoxygenase 1; *PHL11* XM_006352877.2 (*M1AJD4*), MYB family TF; *OMT1* XM_006350136.2 (*M1CLR9*), flavone 3’-O-methyltransferase 1; *STR18* XM_006340685.2 (*M0ZVS2*), rhodanese; *LAC12* XM_006353356.2 (*M1C7N6*), laccase; *LAP2* NM_001318637.1 (*LAP*), leucine aminopeptidase; *LTP1* XM_006345207.2 (*M0ZR50*), non-specific lipid-transfer protein 1; *FT* NM_001287968.1 (*StSP6A*), tuberigen. Detailed information is provided in **Supplementary Table 1**.

The genes *EF1* and *SEC3* were used as housekeeping reference genes for normalization. These genes were selected based on their established stability in Solanaceae species; notably, minimal variation in their C_t_ values was observed across all experimental conditions (MR1, MR8, MST, and NaHS), supporting their suitability as stable controls in this study.

Each sample was subjected to analysis with five biological replicates, and each was tested three times technically during the qPCR process, to determine the relative expression of the genes of interest, applying the 2−ΔΔCT technique (Livak and Schmittgen, 2001).

## 3 Results

### 3.1 MTs development under MST used for the transcriptomic analysis

**Figure 1:**
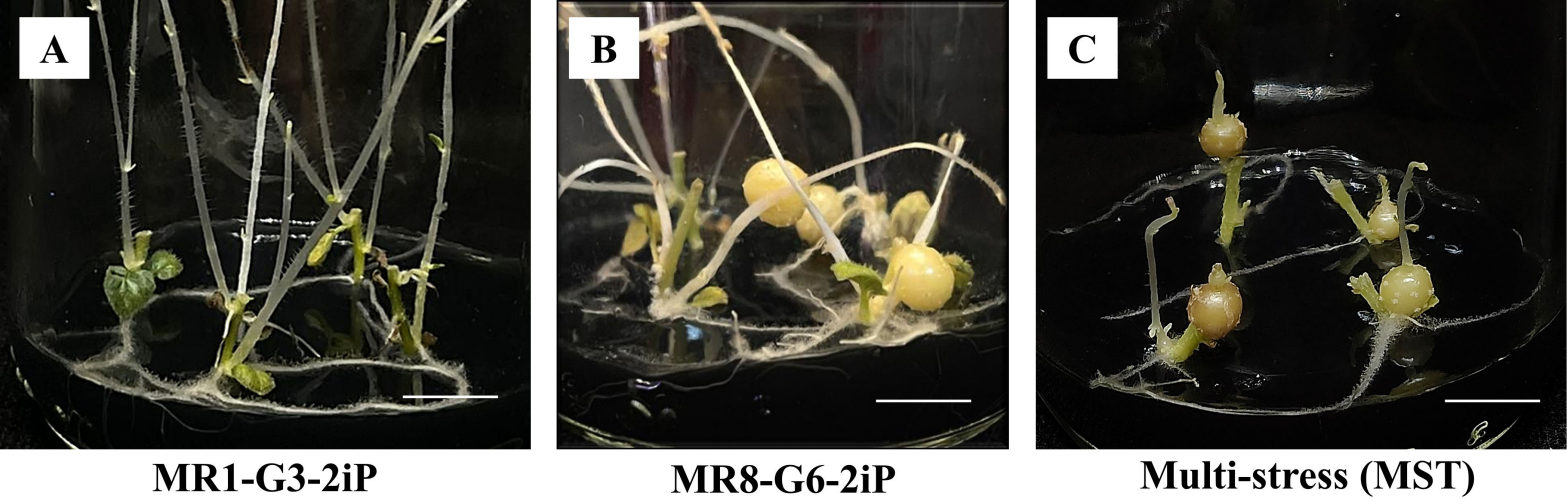
Potato control explants (MR1-G3-2iP) and MTs development derived from stolon explants of potato *S. tuberosum* cv. Alpha, after fifteen days in culture used for the transcriptomic analysis. **(A)** Stolon explants in control medium MR1-G3-2iP; no MTs were observed. **(B)** MTs development in MR8-G6-2iP medium (Sucrose 8%, gelrite 6 g/L; Osmotic stress). **(C)** MTs development under MST treatment (Osmotic, salinity, heat, and cold stress). Scale bars represent 1 cm.

Control stolon explants and MTs used for the transcriptomic analysis were successfully achieved under MR8-G6-2iP medium with high content of sucrose (8%), gelrite 6 g/L, plus 2iP 10 mg/L, according to Valencia-Lozano et al. (2022), as well as MST conditions according to Herrera-Isidron et al. (2024) after two weeks of incubation in darkness (**Figures 1A–C**).

**Figure 2:**
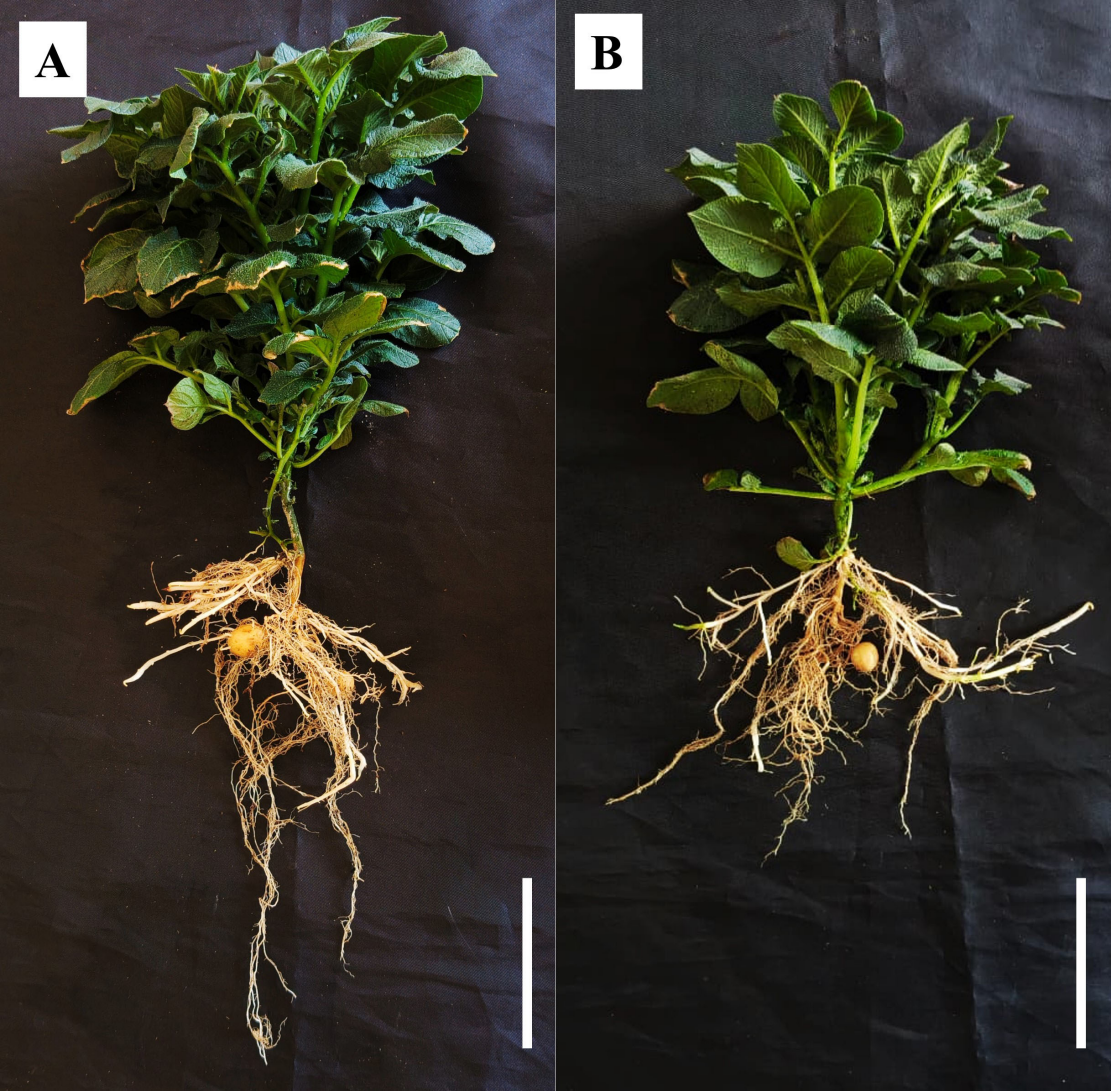
Morphology of potato plants regenerated from *in vitro* MTs, after four months under soil conditions. **(A)** Control potato plant derived from MR8-G6-2iP MTs. **(B)** Plant derived from MST-treated MTs. Scale bar represents 10 cm.

Plant regeneration was successful under both control and MST treatments. On average, two plants were regenerated from each MT. In both treatments, regenerated plants exhibited healthy and fully developed phenotypes, indicating that MST still allowed the formation of a complete and morphologically normal phenotype (**Figures 2A, B**).

After four months under soil conditions, significant differences were observed in foliar area, where the control plants developed 273.1 ± 0.0021^a^ cm² compared to 206.54 ± 7.23^b^ cm² in the MST treatment. Regarding the root system, the control treatment showed a significantly greater root length (30.36 ± 2.69^a^ cm) and root area (152.96^a^ cm²) compared to the MST treatment, which recorded values of 21.64 ± 1.63^b^ cm and 59.42 ± 0.5^b^ cm², respectively (**Table 1**).

**Table 1:** Differences in features among treatments were tested using factorial analysis of variance (ANOVA).

Evaluated feature	Control (± SD)	MST (± SD)
Foliar area (cm^2^)	273.1 ± 0.0021^a^	206.54 ± 7.23^b^
Height (cm)	22.76 ± 2.78	24.13 ± 0.24
Stem diameter (cm)	11.86 ± 2.86	9.6 ± 1.27
Root length (cm)	30.36 ± 2.69^a^	21.64 ± 1.63^b^
Root area (cm^2^)	152.96^a^	59.42 ± 0.5^b^
Tuber diameter (cm)	16.29 ± 7.53	16.3 ± 3.94

Values are means ± standard deviation, and followed by letters when significantly different (P<0.05). Measured with ImageJ software.

In contrast, other features did not show statistically significant differences. The height of the plants remained similar between the control (22.76 ± 2.78 cm) and MST (24.13 ± 0.24 cm) treatments. Likewise, the tuber diameter was nearly identical in both treatments, with values of 16.29 ± 7.53 cm for the control and 16.3 ± 3.94 cm for the MST. While the stem diameter was slightly higher in the control plants (11.86 ± 2.86 cm) compared to the MST ones (9.6 ± 1.27 cm), this difference was not statistically significant.

### 3.2 Effect of NaHS as source of H_2_S in MTs development under MST conditions

To evaluate the effect of hydrogen sulfide (H_2_S) signaling on microtuber (MT) development under MST conditions, explants were treated as previously described, with the addition of the H_2_S donor sodium hydrosulfide (NaHS; Sodium hydrosulfide flakes, CAS 207683, Kandelium México) at two concentrations, 10 µM and 1 mM. These concentrations were selected based on previous studies demonstrating effective modulation of H_2_S signaling in plants (Chen et al., 2011; Lin et al., 2012; Zhang et al., 2020). Explants were incubated for fifteen days under these conditions.

**Figure 3:**
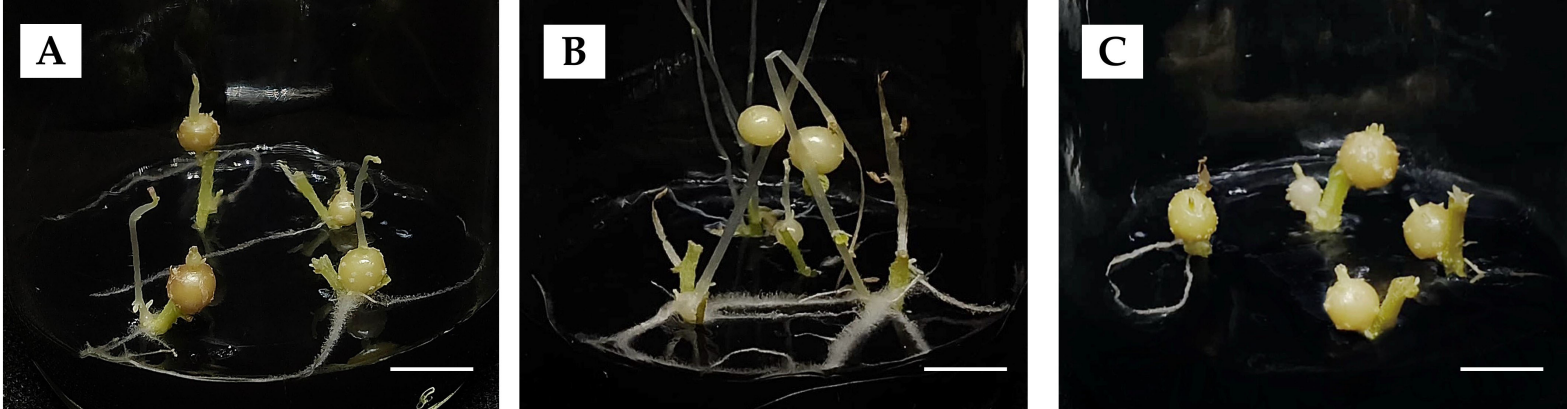
Effect of NaHS as precursor of H_2_S on MTs development under MST conditions. **(A)** MTs development in potato *S. tuberosum* L. cv. Alpha, after fifteen days in culture in MR8-G6-2iP medium supplemented with 50 mM NaCl (salt–osmotic stress) and exposed to 38 °C for 24 h (heat–osmotic stress), and 4 °C for 24 h (cold–osmotic stress). **(B)** MTs development under MST conditions plus 10 µM of NaHS. **(C)** MTs development under MST conditions plus 1 mM of NaHS. Scale bars represent 1 cm.

The application of 1 mM NaHS increased MT area by 11.63% compared with the MST treatment alone, whereas 10 µM NaHS reduced it by 20.19%. These results indicate that H_2_S signaling plays a role in modulating MT development under MST treatment (**Figures 3A–C**).

**Figure 4:**
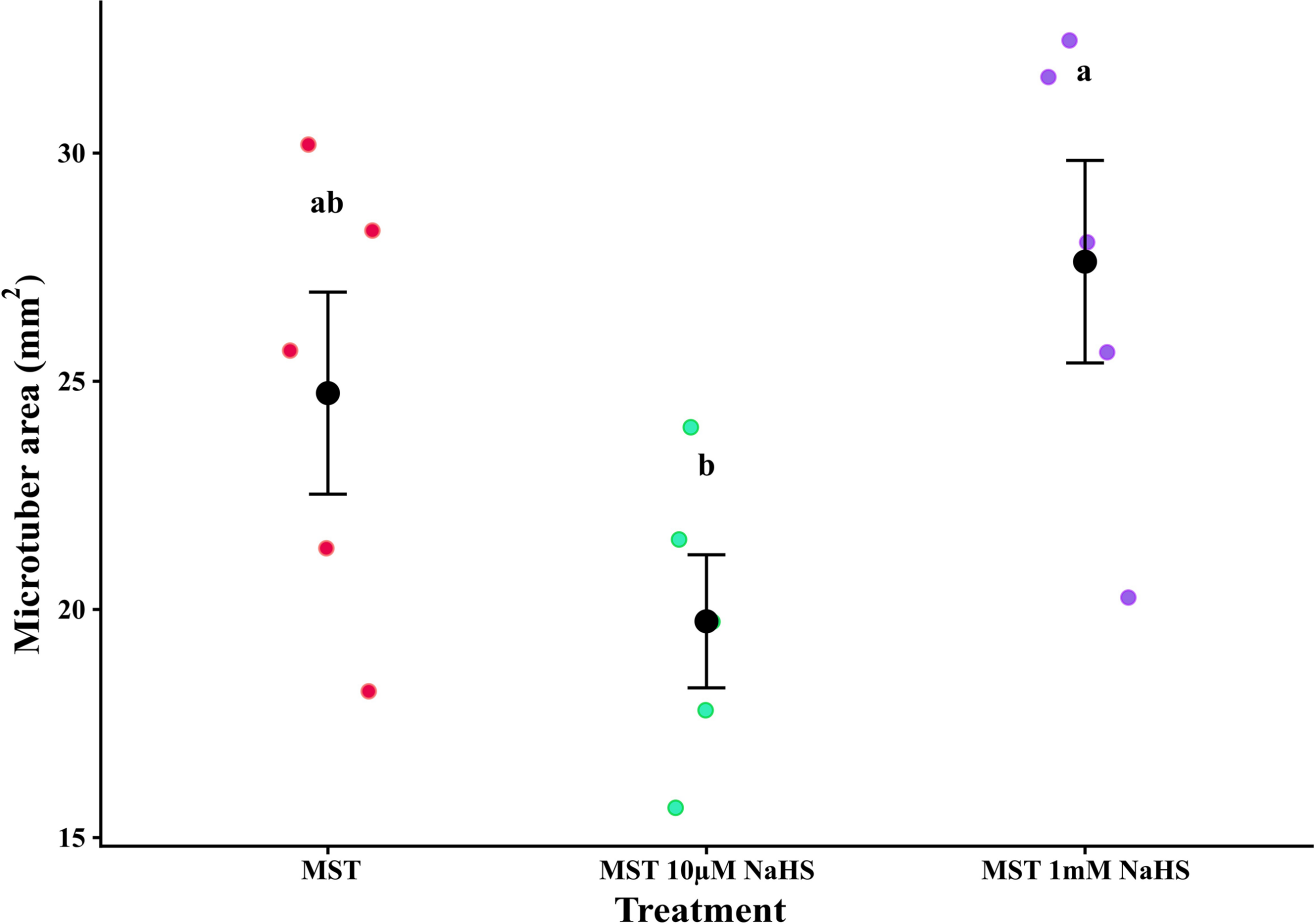
Effect of H_2_S signaling on microtuber development in *Solanum tuberosum*. Individual observations are represented by colored dots, while black circles and error bars indicate the mean ± standard error (SE) (*n* = 5). Different letters indicate significant differences between treatments (Tukey’s HSD, *P* < 0.05).

The magnitude of the treatment effect was large (*η*² > 0.45), indicating that H_2_S signaling accounts for a substantial proportion of the total variance in microtuber development under MST conditions. Pairwise comparisons using Cohen’s *d* confirmed a strong inhibitory effect at 10 µM NaHS and a moderate promotive effect at 1 mM NaHS relative to MST control (**Figure 4**).

### 3.3 Transcriptomic profiles of MTs development under MST conditions

To identify genes involved in MT development under MST conditions, a transcriptome analysis was performed using two comparative datasets.

cDNA libraries were constructed from tissues cultured in MR8-G6-2iP medium, which induces MTs formation, and MR1-G3-2iP medium, which does not induce MTs development. Sequencing was carried out using the Illumina HiSeq 2000 platform, generating a total of 397,834,274 raw reads across six cDNA libraries. These raw reads, representing the unprocessed sequences obtained directly from the sequencer, were mapped to the reference genome to obtain gene-level read counts.

A second comparison was performed between MST-treated samples, which induce MTs formation under combined stress conditions, and MR8-G6-2iP samples, representing MTs induction under standard conditions. For both datasets, gene-level counts were normalized to correct for differences in sequencing depth and library composition, generating normalized counts that allow direct comparison among transcriptomes.

For exploratory analyses, including principal component analysis (PCA) and hierarchical clustering, normalized counts were further transformed using the regularized logarithm (rlog) method to stabilize variance across genes. In total, expression levels were quantified for 22,676 genes across the six cDNA libraries.

Differential expression analyses were conducted independently for the MR1-G3-2iP vs. MR8-G6-2iP and MR8-G6-2iP vs. MST comparisons.

**Figure 5:**
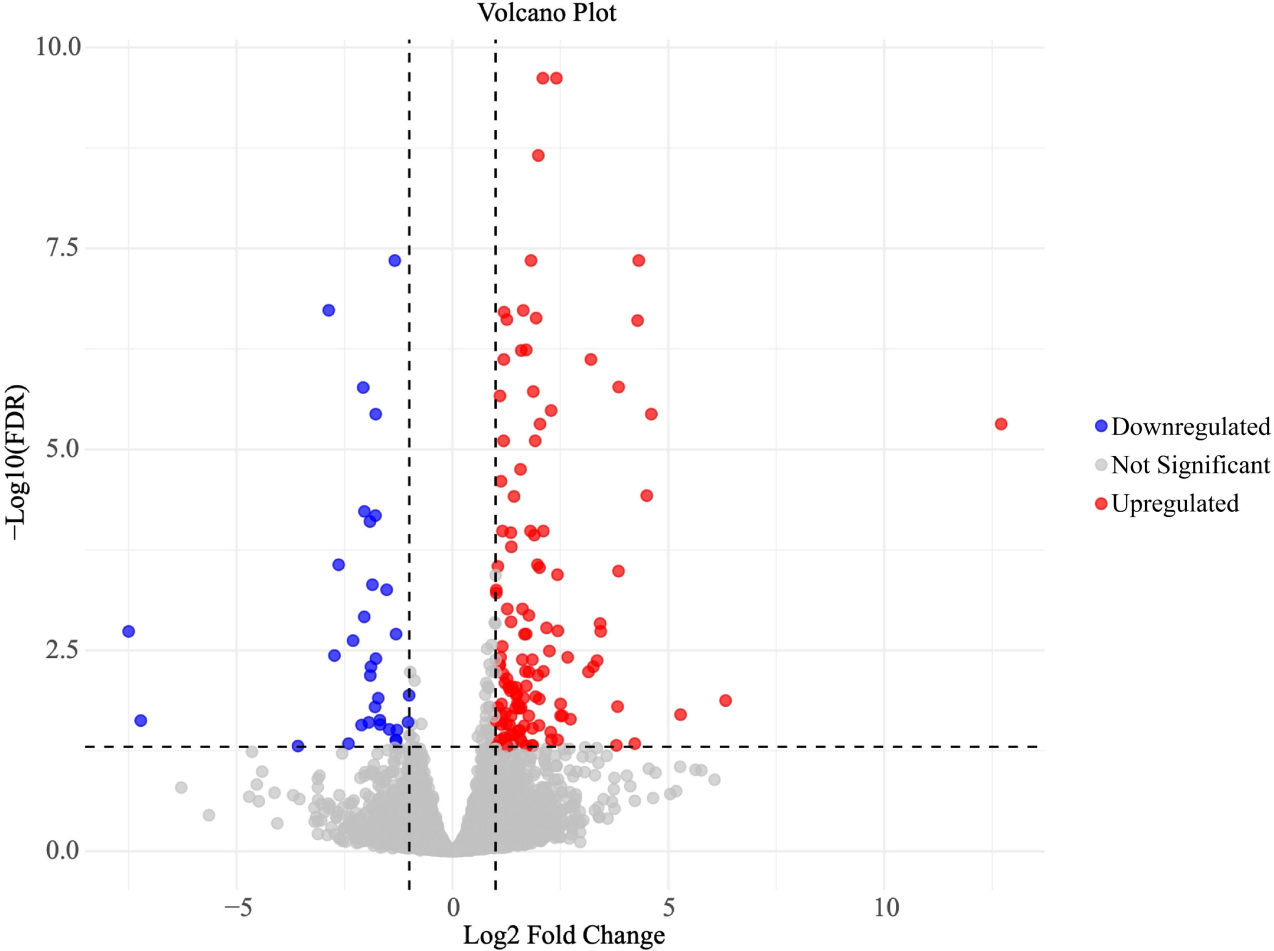
Volcano plot of differentially expressed genes (DEGs) in the MR8-G6-2iP vs. MST comparison. The plot displays the distribution of genes according to their log2 Fold Change and statistical significance as −log10 FDR. Genes meeting the significance threshold (Log2FC ≥ 1.5 and FDR < 0.05) are represented by red dots (upregulated) and blue dots (downregulated), while non-significant genes are shown in gray. Genes with higher statistical significance are located toward the top of the plot.

To visualize the distribution of transcriptomic changes during MST induction, a volcano plot was generated for the MR8-G6-2iP vs. MST comparison (**Figure 5**). In this specific contrast, only a subset of the total pool reached the significance threshold (log2FC ≥ 1.5, adjusted p-value < 0.05), as represented by the red and blue points.

Beyond this individual comparison, the union of both datasets revealed a total of 1,475 upregulated genes. Furthermore, a total of 2,046 shared DEGs (common to both comparisons) were identified, of which 1,212 were upregulated and 834 were downregulated. A summary of mapping statistics for each sample is shown in **Figure 5**.

Gene Ontology (GO) enrichment analysis was performed on the 2,046 shared DEGs. Initially, a total of 1,212 GO term annotations were identified across the biological process, molecular function, and cellular component categories for these genes. Subsequent enrichment analysis (FDR < 0.05) identified that both up- and downregulated genes were associated with broad biological processes, such as protein phosphorylation, transcriptional regulation, transmembrane transport, carbohydrate metabolic processes, and proteolysis.

Among the upregulated genes, significantly enriched biological processes included microtubule-based movement, translation, fatty acid biosynthetic process, lipid transport, DNA replication, glycolysis, positive regulation of transcription, cell wall modification, DNA repair, defense response, carboxylic acid metabolic process, protein dephosphorylation, glutathione metabolic process, and amino acid metabolic process.

**Figure 6:**
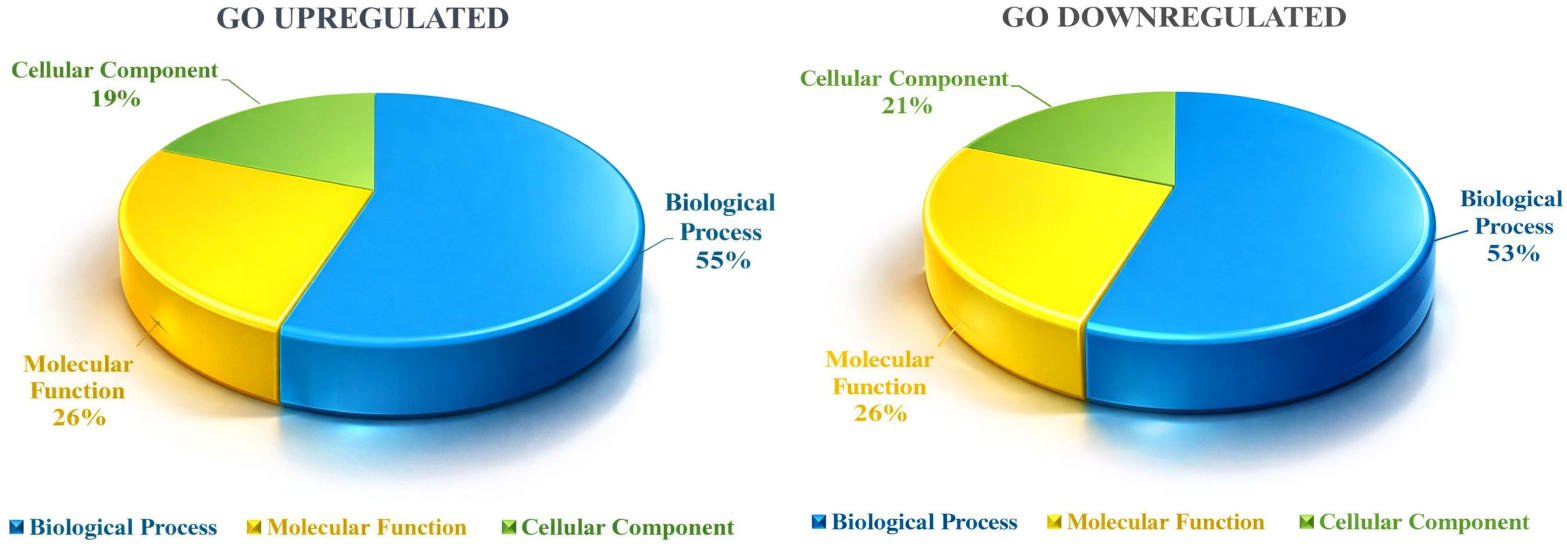
Enriched biological processes regulated during potato *S. tuberosum* L. cv Alpha MTs development under MST conditions.

In contrast, downregulated genes were primarily associated with photosynthesis-related processes, including light harvesting, cell wall biogenesis, cellular glucan metabolic process, and xyloglucan metabolic process (**Figure 6**).

**Figure 7:**
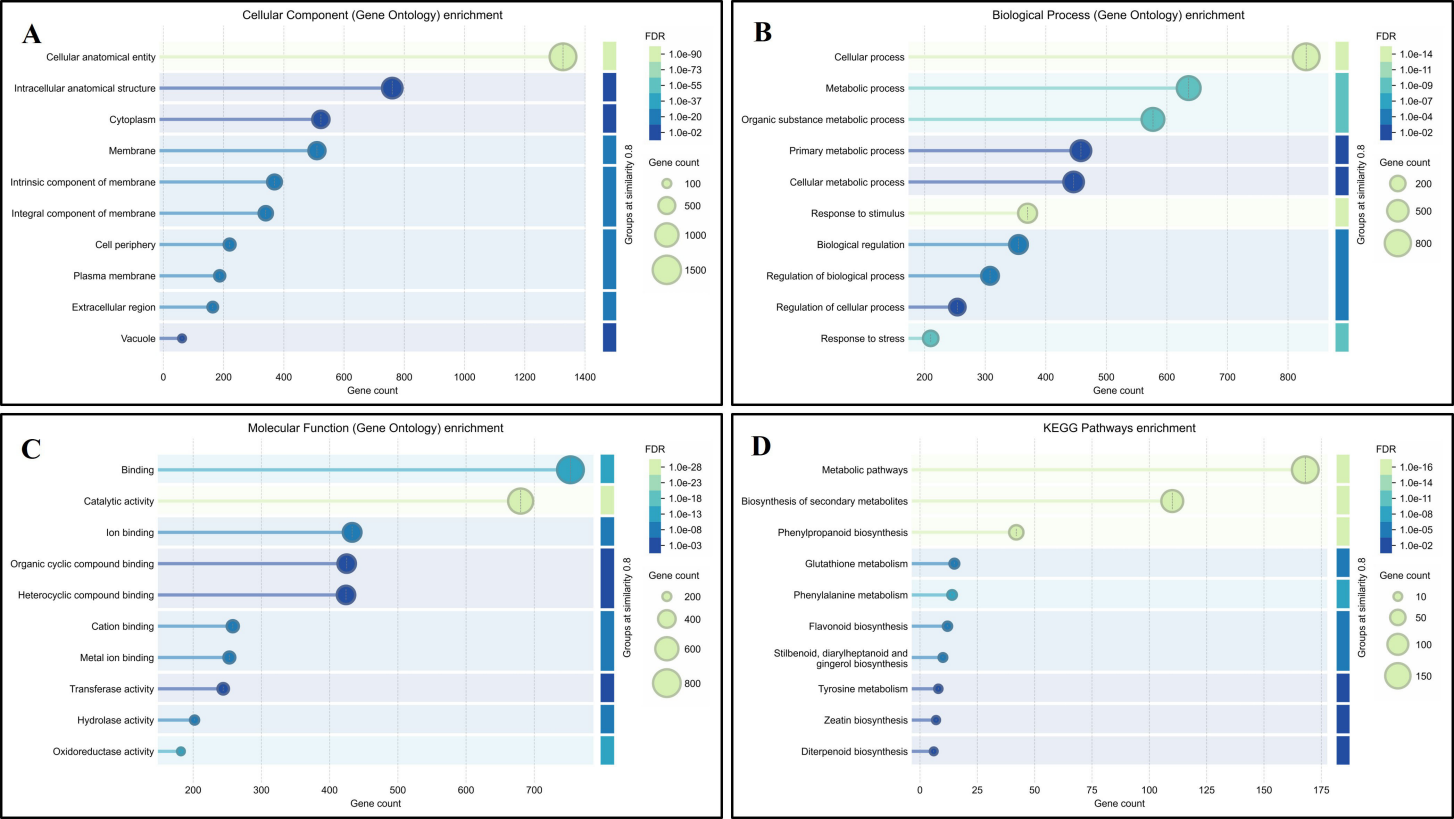
Functional enrichment analysis of DEGs associated with MT development in *Solanum tuberosum* (L) cv. Alpha under MST conditions. **(A)** GO cellular component enrichment. **(B)** GO biological process enrichment. **(C)** GO molecular function enrichment. **(D)** KEGG pathway enrichment analysis.

GO enrichment analysis of the 2,046 shared DEGs revealed a predominance of biologically informative processes associated with stress adaptation and developmental reprogramming. Among the most significantly enriched biological processes, were oxidation–reduction processes, phenylpropanoid biosynthesis, cell wall modification, lipid metabolic processes, and hormone-mediated signaling pathways. These categories are consistent with the transcriptional reconfiguration required for MT development under sequential multi-stress conditions. Additionally, enrichment of microtubule-based movement and DNA replication-related terms suggests active cellular restructuring and proliferation during MT formation (**Figures 7A, B**).

In the KEGG pathways category, the most significantly enriched pathways included Metabolic pathways, Biosynthesis of secondary metabolites, Phenylpropanoid biosynthesis, Glutathione metabolism, Phenylalanine metabolism, Flavonoid biosynthesis, Stilbenoid, Diarylheptanoid and Gingerol biosynthesis, Tyrosine metabolism, Zeatin biosynthesis, and Diterpenoid biosynthesis (**Figure 7D**). Statistical significance was assessed using FDR-adjusted p-values below 0.05, indicating robust enrichment of these pathways in response to MST treatment.

### 3.4 Protein-protein interaction analysis

Transcriptomic analysis of potato MTs development under MST revealed 1,475 upregulated genes resulting from the union of both experimental comparisons (MR1-G3-2iP vs. MR8-G6-2iP and MR8-G6-2iP vs. MST), that assemble into a 317-gene PPI network using the STRING-database v12.0 organized into 17 modules (**Supplementary Table 2**). This reduction (78.5% of DEGs) occurred because the network construction prioritized functional interactions and stringent sequence identity with *A. thaliana* (≥ 60%), ensuring the network is grounded in validated biological pathways.

Functional characterization of the 1,158 non-interacting proteins excluded from the MST PPI network revealed that many belong to categories strongly associated with stress adaptation and signaling in Solanaceae and other crop species. The excluded fraction included hydrolase-related proteins, Cytochrome P450 superfamily members, oxidoreductases, leucine-rich repeat (LRR) proteins, transcriptional regulators, membrane-associated proteins, and extracellular signaling components. These functional groups are widely implicated in abiotic stress perception, redox homeostasis, membrane remodeling, receptor-mediated signaling, and transcriptional reprogramming under combined stress conditions.

Particularly relevant was the enrichment of 220 cell periphery-associated proteins, including receptor-like kinases, peptidases, anion channels, and pentatricopeptide-containing proteins involved in stress sensing and signaling. Furthermore, it contained 340 integral membrane proteins, essential for membrane dynamics and transport regulation during stress adaptation, as well as 160 extracellular region-associated proteins linked to extracellular matrix remodeling and intercellular communication under stress conditions.

These categories often evolve rapidly and may exhibit lower sequence conservation outside Solanaceae lineages, making them more likely to be underrepresented in *Arabidopsis*-centered interaction databases. Therefore, the excluded proteins may contain lineage-specific regulatory components relevant to potato stress adaptation that conserved orthology-based PPI reconstruction does not capture.

This network (0.500 threshold) includes a mixture of interaction confidence levels: approximately 29.8% high-confidence interactions (>0.700) and 70.2% medium-confidence interactions (>0.500-0.700). This reflects an inclusive strategy aimed at capturing both experimentally supported and predicted interactions. Notably, key hub proteins and major functional modules remained conserved when higher confidence thresholds (0.700–0.900) were applied, supporting the stability of the core network architecture despite its predictive nature.

**Figure 8:**
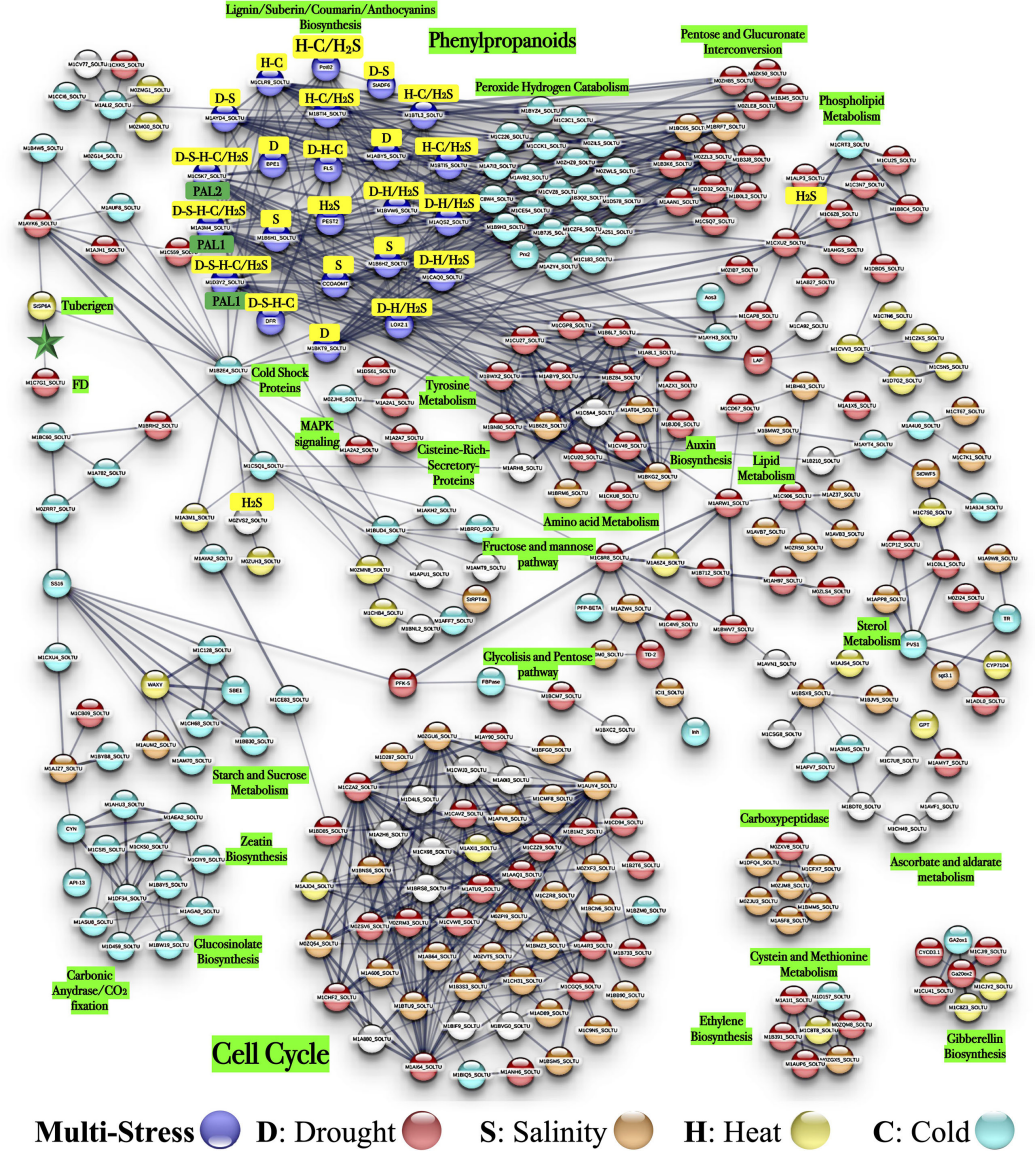
PPI network of upregulated genes associated with MT development in *Solanum tuberosum* L. under MST conditions. The network was generated using the STRING database v12.0 with confidence score threshold of 0.500.

The identified modules were associated with MST-responsive proteins, phenylalanine metabolism, phenylpropanoid metabolism, hydrogen peroxide catabolism, pentose and glucuronate interconversion, phospholipid metabolism, lignin degradation, lipid metabolism, cell cycle, starch and sucrose metabolism, amino acid metabolism, ascorbate and aldarate metabolism, carboxypeptidase, sterol, auxin, ethylene, gibberellin, and zeatin/carbonic anhydrase/glucosinolate biosynthesis (**Figure 8**).

### 3.5 Upregulated TFs under MST conditions

**Figure 9:**
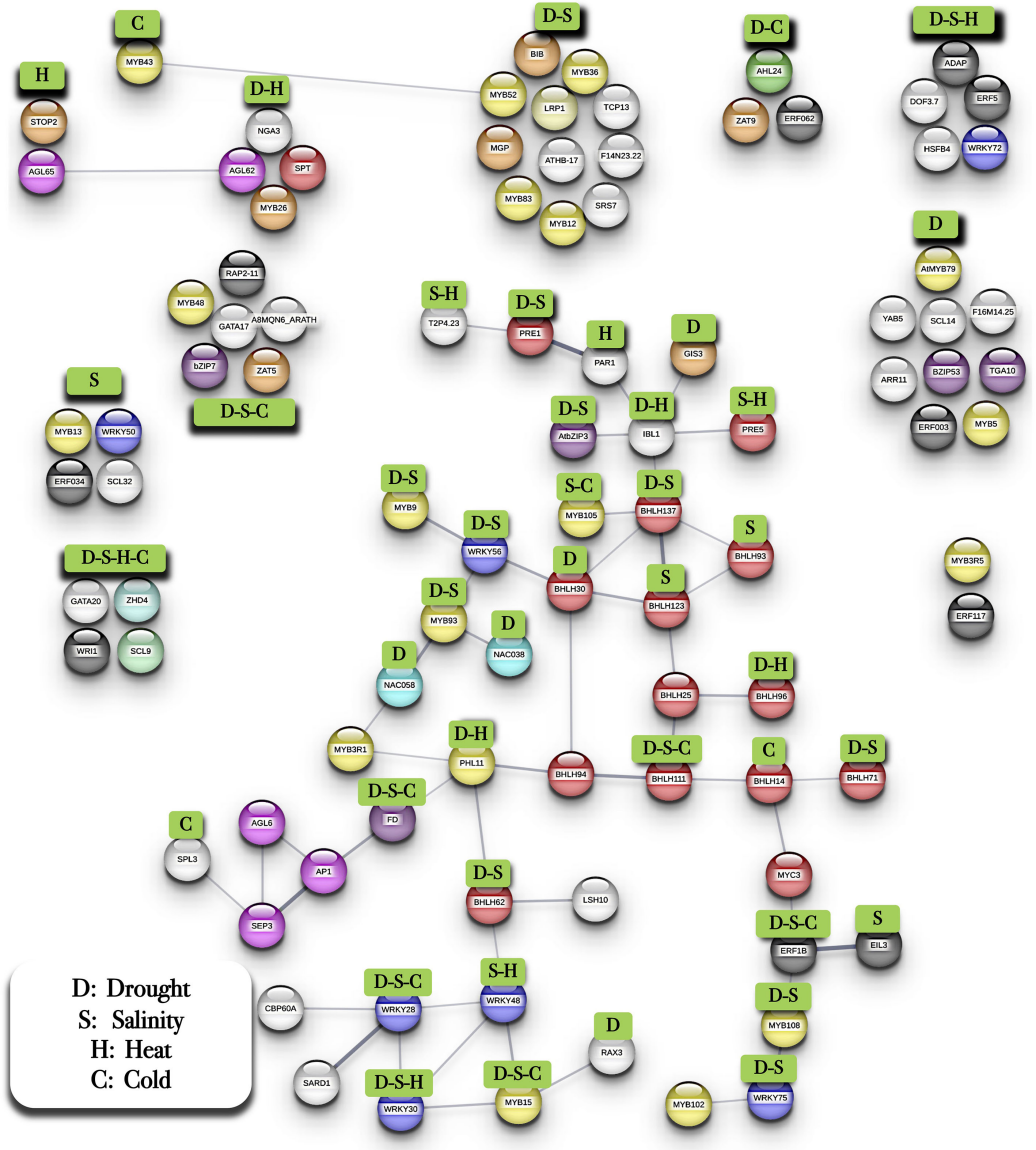
PPI network of upregulated TFs involved in potato MTs development under MST (osmotic, salinity, cold and heat) conditions, derived from the STRING database v12.0 of potato *S. tuberosum* L. from the transcriptomic-wide analysis with confidence (0.400).

Overall, 96 upregulated TFs were found in the transcriptomic-wide analysis of MTs development under MST conditions. The upregulated TFs belong to SANT/Myb domain (23), bHLH (15), AP2/ERF (9), Zinc finger C2H2-type (7), WRKY (7), bZIP (5), MADS-box (5), GATA (2), Homeobox-like (1), SARD1 (1) involved during potato MST responses. The analysis of combined and individual stress responsive TFs revealed that 22 of them are involved in responses to three and four combined stresses, whereas 56 involved in one or two combined stresses (**Figure 9**).

### 3.6 Comparative and evolutionary perspective on lignin-related TFs in potato

The cultivated potato, *S. tuberosum* L., conserves the canonical ERF–NAC–MYB–bZIP regulatory framework governing lignin biosynthesis in model and crop species such as *Arabidopsis thaliana, Oryza sativa*, and *Malus domestica*; however, it exhibits a notable expansion of stress-responsive ERF, WRKY, and MYB modules, reflecting a structurally divergent regulatory architecture characterized by enhanced integration of environmental signaling pathways (Choi et al., 2023).

Our analysis reveals that potato displays a broad and modular TFs repertoire to govern lignin biosynthesis, characterized by the recruitment of MYB (n = 18), bHLH/MYC (n = 13), ERF/AP2 (n = 15), WRKY (n = 7), NAC (n = 3), bZIP (n = 3), and C2H2 zinc-finger repressors (n = 4). The presence of such a diverse array of regulators suggests that lignin deposition during MT development is not merely a programmed developmental event, but a highly adaptative process, indicating a sophisticated integration of environmental signaling layers, where stress-responsive factors and metabolic tuners converge to fine-tune secondary cell wall reinforcement under multi-stress conditions.

Evolutionarily conserved regulators—including MYB83, MYB52, MYB43, ERF5, RAV1/2, and ZAT family members—indicates preservation of a core lignification module across angiosperms. The formation of protective barriers like the biopolymers; lignin, suberin, cutin and sporopollenin that reinforce and waterproof by the polysaccharide-based cell wall were critical innovations of land plant evolution (Kriegshauser et al., 2021). Nevertheless, the numerical enrichment of stress-responsive TFs in potato points to lineage-specific diversification likely associated with adaptation to fluctuating environmental conditions, especially water deficit. Similar patterns have been described in drought-tolerant crops such as rice and apple, where enhanced lignin deposition in vascular tissues correlates with improved drought resilience.

### 3.7 PCA analysis of TFs during MTs development under MST

**Figure 10:**
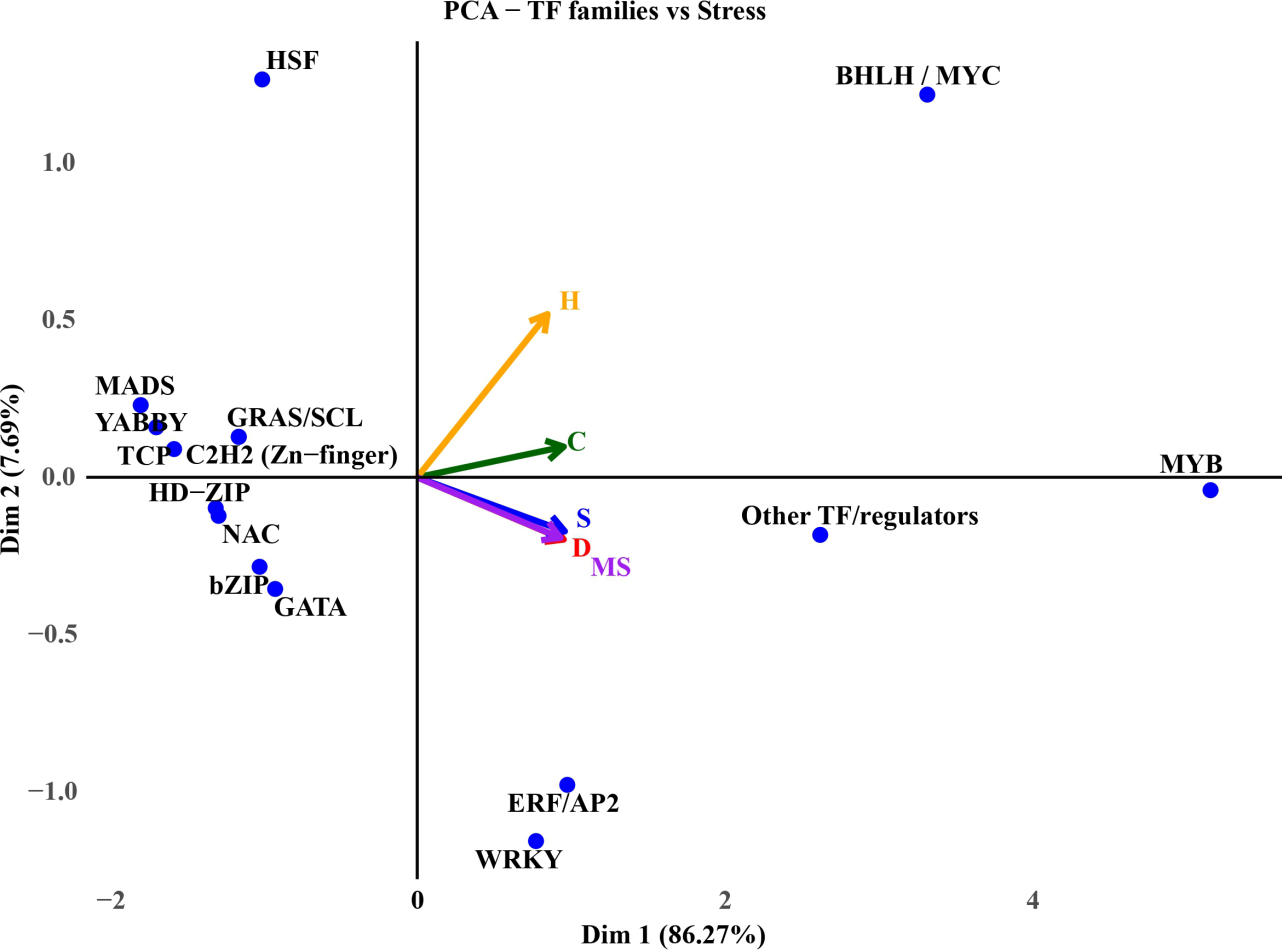
Principal component analysis (PCA) from the relative gene expression under MST during MTs development of potato *S. tuberosum* L. cv Alpha. The TF families with the highest variance were the MYB followed by the BHLH/MYC and other TFs.

Principal Component Analysis (PCA) was conducted on TFs family expression profiles in the cultivated potato, *S. tuberosum* L., under MST conditions, including drought, salinity, heat, cold, and combined treatments (**Figure 10**). Dimension 1 (Dim.1) explained 86.3% of the total variance, representing the dominant axis of transcriptional reprogramming, whereas Dimension 2 (Dim.2) accounted for 7.7%, reflecting secondary regulatory differentiation.

The highest contributions to Dim.1 were observed for MYB (42.86), bHLH/MYC (16.98), and other TFs/regulators (10.60), indicating that these families primarily drive global transcriptional variation across stress conditions and may function as central integrators of MST responses. In contrast, MADS-box (4.99), YABBY (4.44), TCP (3.86), and HD-ZIP (2.65) displayed lower contributions, suggesting more restricted or context-dependent regulatory roles.

Along Dim.2, HSF (27.78) and WRKY (23.22) showed the strongest contributions, highlighting their involvement in specialized or stress-specific regulatory pathways that differentiate particular stress combinations during MTs development.

Collectively, these results reveal a hierarchical transcriptional structure in which a limited set of TF families dominates global stress-associated variation, while others contribute to finer regulatory tuning. This framework enables prioritization of candidate TFs for functional validation and targeted strategies aimed at improving MST resilience during MTs development (**Table 2**).

**Table 2:** TFs families, stress categories, functional evidence, mutant analysis of the upregulated TFs under MST during MTs development of potato *S. tuberosum* L. cv Alpha.

TF family	Arabidopsis homologous (*S. tuberosum* ID)	Stress categories	Functional evidence	Mutant evidence	Citas
*MYB*	*AtMYB79 (M1CLG8), MYB102 (M1AF71), MYB105 (M1CPY7), MYB108 (M1A032), MYB12 (M1AI58), MYB13 (M1BS18), MYB15 (M1C5C0), MYB26 (M1DDW8), MYB36 (M0ZPG2), MYB3R1 (M0ZTQ2), MYB3R5 (M1AHL2), MYB43 (M1C651), MYB48 (M1AQV3), MYB5 (M1A577), MYB52 (M1CBE0)*, *MYB83 (M1A9C0), MYB9 (M1BH84), MYB93 (M1A7L8)*	D, S, H, C, MS, Ox (6)	Regulation of phenylpropanoid metabolism, lignification, and stress- responsive transcription	Multiple MYB loss-of-function lines alter drought/salt tolerance	Li et al., 2019a; Ren et al., 2023
*ERF/AP2*	*ERF003 (M1CGW6), ERF034 (M1CGG5), ERF062 (M1D3E8), ERF1B (M1B0S4), ERF5 (M1CHB7), RAP2-11 (M1BFD0), ERF117 (M1B5V5)*	D, S, H, C, MS (5)	ABA/ethylene-mediated multi-stress signaling	ERF mutants affect drought and flooding responses	Jian et al., 2020; Park et al., 2022b; Zhang et al., 2023; Luo et al., 2025; Velivelli et al., 2015; Liu et al., 2023; Moffat et al., 2012; White et al., 2017
*bHLH / MYC*	*BHLH111 (M1BCN6), BHLH123 (M1A253), BHLH137 (M1CKP3), BHLH14 (M1A3Q9), BHLH25 (M1A291), BHLH30 (M1AN65), BHLH62 (M0ZZX3), BHLH71 (M1BHA5), BHLH93 (M1A7J7), BHLH94 (M0ZPW7), BHLH96* *(M1CS96), MYC3 (M1A599)*	D, S, H, C, MS (5)	Integration of ABA, JA and redox signaling	Mutants alter abiotic stress resilience	Ren et al., 2023; Cao et al., 2025; Gong et al., 2018; Fang et al., 2024; Liu et al., 2021
*WRKY*	*WRKY28 (M1AI80), WRKY30 (M1BRE7), WRKY48 (M1CLH8), WRKY50 (M1AEP5), WRKY56 (M1AWU5), WRKY72 (M1A9L5), WRKY75 (Q8H9D7)*	D, S, H, MS (4)	Stress amplification and defense crosstalk	WRKY75 mutants affect phosphate and drought signaling	Li et al., 2019b; Li et al., 2020; Balfagón et al., 2024; Zhu et al., 2025; Zou et al., 2019; Gao et al., 2011
*NAC*	*NAC038 (M1B6X0), NAC058 (M1A3A8), SARD1 (M1A3B9)*	D, MS (2)	Secondary wall formation and stress regulation	NAC mutants alter drought tolerance	—
*bZIP*	*AtbZIP3 (M0ZLT8), BZIP53 (M0ZVK3), bZIP7 (M1A3K4)*	D, S, H, MS (4)	ABA-dependent transcriptional control	bZIP mutants affect stress- responsive gene activation	Dietrich et al., 2011; Wang et al., 2024; Liu et al., 2023
*C2H2 (ZAT)*	*ZAT5 (M1D2E8), ZAT9 (M1CA32)*	D, S, C, MS (4)	Zinc-finger redox-responsive regulation	ZAT mutants alter ROS tolerance	Wang et al., 2022; Fan et al., 2021; Meena et al., 2024; Sehar et al., 2025
*GRAS / SCL*	*SCL14 (M0ZPY5), SCL32 (M1CH56), SCL9 (M1AHK3)*	D, S, H, MS (4)	Detoxification and stress adaptation	SCL mutants affect stress acclimation	Fode et al., 2008; Zhao et al., 2021; Wang et al., 2019
*HSF*	*HSFB4 (M1ADP6)*	D, S, H, MS (4)	Heat and oxidative stress response	HSF mutants impair thermotolerance	—
*TCP*	*TCP13 (M1CIV8)*	D, S (2)	Growth–stress balance regulation	Limited functional mutant data	—
*GATA*	*GATA17 (M0ZQP5), GATA20 (M1B993), GIS3 (M1B5S5)*	D, S, H, MS (4)	Light and nitrogen-responsive stress integration	GIS mutants alter developmental stress plasticity	—
*MADS-box*	*AGL6 (M0ZGN7), AGL62 (M0ZKW1), AGL65 (M1BQ16), AP1 (M1CR50)*, *SEP3 (M1BZC3)*	Dev, D, MS (3)	Developmental control linked to stress adaptation	SEP3/AGL mutants alter flowering and stress responses	Kaufmann et al., 2009; Jing et al., 2024; Dezar et al., 2011; Sun et al., 2024
*YABBY*	*YAB5 (M0ZT20)*	Dev, MS (2)	Organ identity and stress-associated morphogenesis	Limited mutant data	—
*HD-ZIP*	*ATHB-17 (M1CZW3), ZHD4 (M1BUX1)*	D, S, H, MS, Ox (5)	Redox and abiotic stress signaling	ATHB17 mutants alter oxidative tolerance	—
Other TF / Regulators	*ADAP (M1B436), FD (M1C7G1), STOP2 (M0ZHX7), WRI1 (M1CML9), AHL24 (M1BHG3), ARR11 (M1AVF1), NGA3 (M1A594), PAR1 (M1BGJ8), SPL3 (M1D8X0), SPT (M0ZXR3), IBL1 (M1AS43), LRP1 (M1AK47), LSH10 (M1B8U2), MGP (M1BPB7), PRE1 (M0ZLZ0), PRE5 (M0ZV09), PHL11* *(M1AJD4), RAX3 (M1A1M3), T2P4.23 (M1CIW7), F14N23.22 (M0ZYX8)*,	D, S, H, C, Dev, MS (6)	Hormonal integration, developmental signaling, redox and metabolic control	Heterogeneous mutant evidence	Lee et al., 2009; Kobayashi et al., 2014; Yang et al., 2025; Enomoto et al., 2019

**Figure 11:**
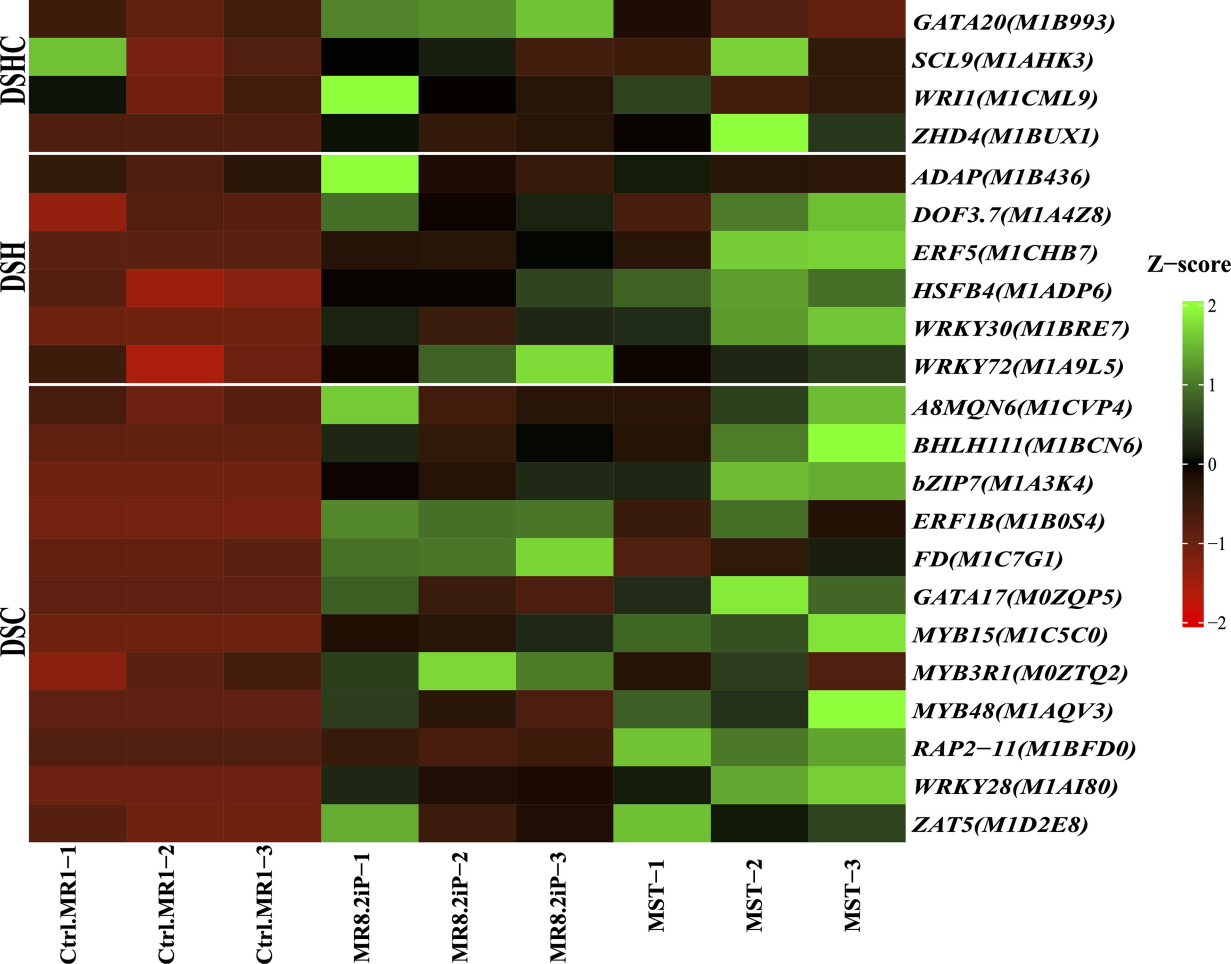
Heat map displaying of the upregulated TFs involved in MST (in response to DSHC, DSH and DSC) during potato MTs development in darkness; the upregulation levels are shown in Log2. D,drought; S, salt; H, heat; C, cold.

The heat map displaying the upregulated TFs involved in MST (in response to DSHC, DSH and DSC) during potato MTs development in darkness; the upregulation levels are shown in Log2. D (drought), S (salt), H (heat), C (cold) (**Figure 11**).

**Figure 12:**
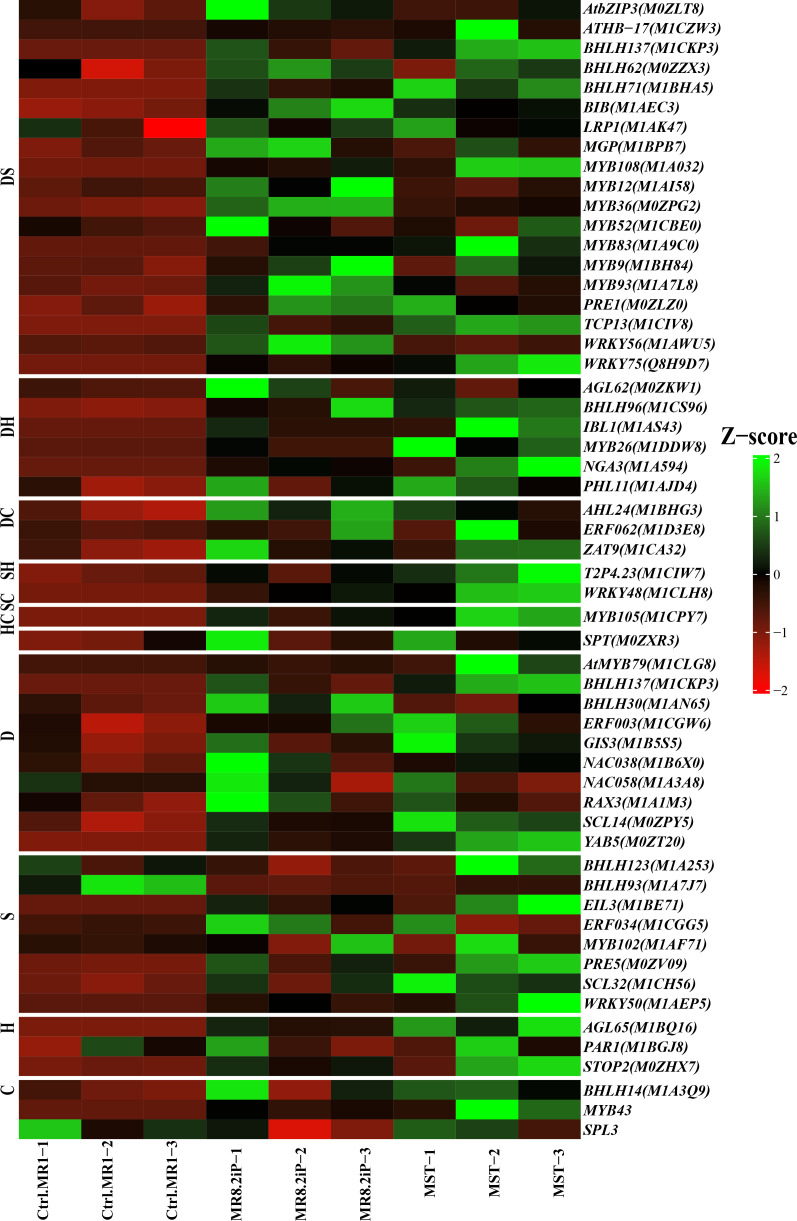
Heat map displaying of the upregulated TFs involved in MST (in response to different combined stresses) during potato MTs development in darkness; the upregulation levels are shown in Log2. D, drought; S, salt; H, heat; C, cold.

The heat map displaying the upregulated TFs involved in MST in response to different combined and unique stresses during potato MTs development in darkness; the upregulation levels are shown in Log2. D (drought), S (salt), H (heat), C (cold) (**Figure 12**).

A comprehensive list of the upregulated TFs found in MTs development under MST conditions is present in **Table 2**.

### 3.8 Validation of DEG by qPCR of MST response (relative expression-qPCR)

**Figure 13:**
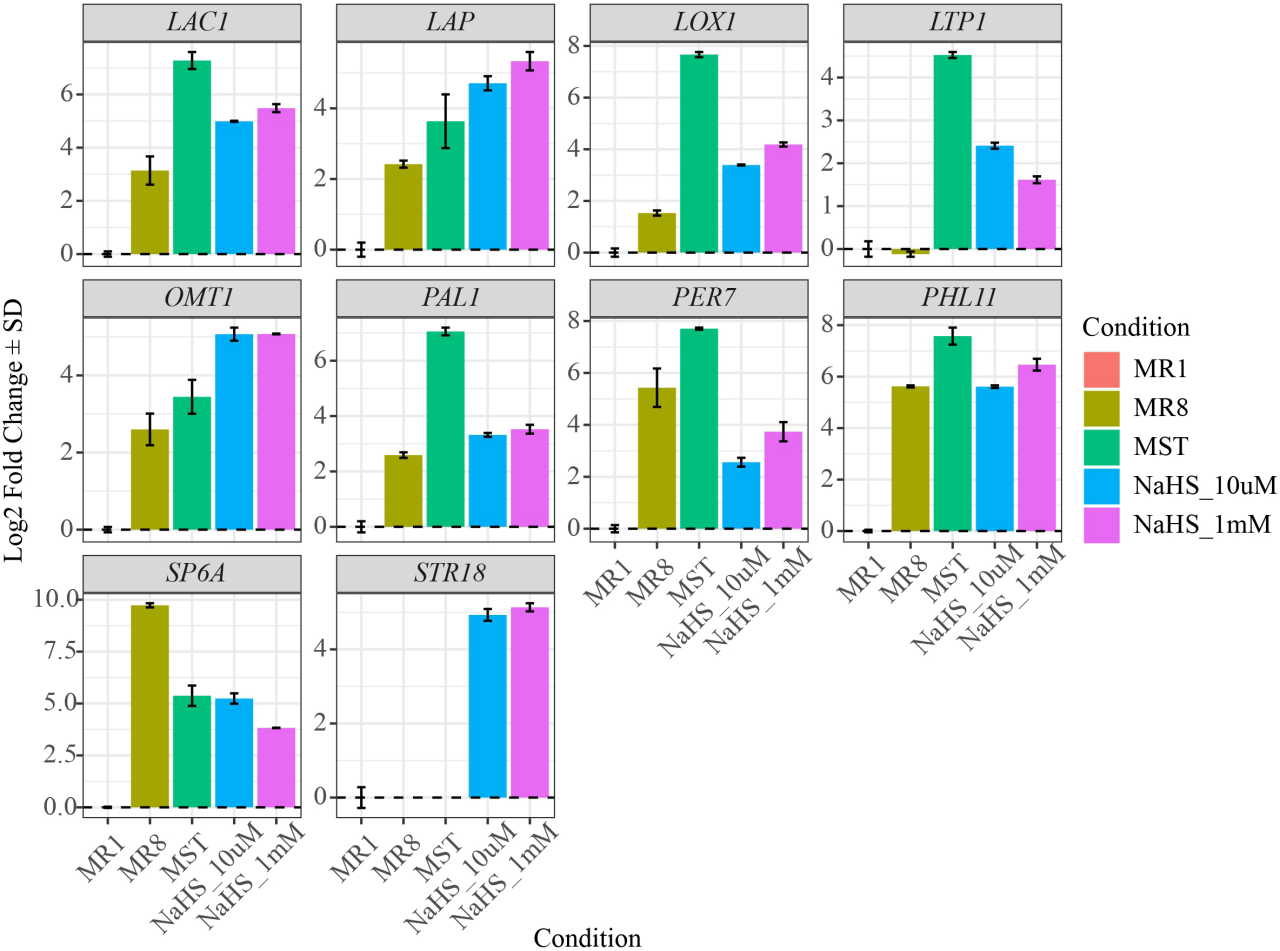
Quantitative PCR analysis of the stolon explants producing MTs of 10 selected genes under MR1, MR8, MST, NaHS 10 µM and NaHS 1 mM. Relative expression estimation levels are represented in Log2-Fold Change.

Validation of the transcriptomic-wide analysis was made by selecting 10 DEG and analyzing their regulation by quantitative reverse transcription PCR (qRT-PCR), using the primers described in **Supplementary Table 1**. The results are shown in **Figure 13**, indicating that the values are consistent with those obtained in the transcriptomic-wide analysis.

The qPCR validation supported the transcriptomic trends observed under MST conditions. As expected, all genes showed log2FC = 0 in MR1, which was used as calibrator.

Under MR8, a subset of genes was strongly induced, particularly *SP6A* (9.73), *PER7* (5.43), and *PHL11* (5.62), indicating early activation of developmental and redox-related pathways.

In contrast, the MST treatment triggered a broader and more coordinated transcriptional responses, with consistently high induction of *PER7 (M1B2E4)* (7.70), *LOX1 (M1AQS2)* (7.66), *PHL11 (M1AJD4)* (7.58), *LAC1 (M1C7N6)* (7.27), *PAL1 (M1D3Y2)* (7.05), *LTP1 (M0ZR50)* (4.4), *OMT1 (M1CLR9)* (4.4). The strong activation of *StSP6A* (5.38 under MST) confirms its central role as a developmental integrator under combined stress, reinforcing the RNA-seq network prediction that positioned the *StSP6A* module as a regulatory hub linking tuber initiation with stress-responsive metabolism.

This interpretation is consistent with previous reports showing that *StSP6A* overexpression enhances tuberization even under combined heat–drought conditions (Navarro et al., 2011; Lehretz et al., 2021), supporting its function as a resilient developmental integrator.

Notably, genes associated with phenylpropanoid metabolism (*PAL1 (M1D3Y2), OMT1 (M1CLR9)*), oxylipin biosynthesis (*LOX1 (M1AQS2)*), redox regulation (*PER7 (M1B2E4)*), sulfur metabolism (*STR18 (M0ZVS2)*), and cell wall remodeling (*LAC1 (M1C7N6), LAP2 (LAP)*) were consistently upregulated under MST and H_2_S treatments (NaHS 10 µM and 1 mM).

The induction of *PER7 (M1B2E4)* aligns with the established role of class III peroxidases in ROS modulation and structural remodeling (Passardi et al., 2005; Baxter et al., 2014), while the activation of *PAL1 (M1D3Y2)* and *OMT1(M1CLR9)* supports the involvement of phenylpropanoid pathways as adaptive metabolic interfaces under stress (Bhardwaj et al., 2013; Kriegshauser et al., 2021).

Likewise, the strong upregulation of *LOX1* (*M1AQS2*) is consistent with oxylipin-mediated stress signaling, and the responsiveness of *STR18 (M0ZVS2)* under NaHS treatments reinforces the integration of sulfur metabolism within redox–gasotransmitter networks.

The NaHS treatment as precursor of H_2_S induced a coordinated response pattern similar to MST treatment, particularly at 1 mM, where *PHL11 (M1AJD4)* (6.46), *LAC1 (M1C7N6)* (5.48), *LAP2 (LAP)* (5.33), *STR18 (M0ZVS2)* (5.13), and *OMT1(M1CLR9)* (5.07) showed strong activation. Collectively, these findings support the idea observed in the MST PPI network, that hydrogen sulfide functions as an integrative signaling node linking redox homeostasis, phenylpropanoid remodeling, and developmental regulation. Thus, the qPCR results not only validate the RNA-seq data but also consolidate a mechanistic framework in which MTs development under MST is embedded within a highly interconnected regulatory network integrating developmental signaling, metabolic reallocation, and redox–gasotransmitter crosstalk.

## 4 Discussion

Potato crops increasingly face concurrent osmotic and salinity constraints, together with temperature fluctuations. This complexity requires network-level reconstruction to identify integrated developmental-stress control states that drive production losses and compromise tuber quality. MST conditions impose a level of regulatory demands that cannot be inferred from single-stress paradigms.

In this context, we acknowledge that the lack of single-stress controls precludes the attribution of specific responses to individual stress factors; however, our study was designed to characterize the transcriptional landscape of a defined sequential stress regime during microtuberization.

The concurrent exposure to drought, salinity, heat, and cold stress during potato MTs development triggered coordinated transcriptional reprogramming, with 1,475 upregulated genes and the assembly of 317 of these into a highly interconnected PPI network. This connectivity pattern is consistent with a systems-level shift in which developmental control, metabolic allocation, redox homeostasis, and structural remodeling operate as coupled layers rather than independent stress outputs.

While the final PPI assembly focused on 317 genes, we acknowledge that the exclusion of the remaining 78.5% of upregulated DEGs highlights a current challenge in Solanaceae bioinformatics. Although their omission is a limitation, it ensures that the identified modules, represent highly reliable regulatory hubs rather than unsupported inferences based on low-confidence homology.

Furthermore, these observed transcriptional changes may reflect both the amplification and reprogramming of pre-existing osmotic and hormonal signaling pathways, rather than entirely *de novo* responses.

This system-level coupling is substantiated by three convergent findings in our dataset. First, MST induces broad transcriptional control, with 96 upregulated TFs spanning multiple families (**Figure 8****;** **Table 2**). Second, the STRING network organizes into 17 functional modules, including a hormone-related module linked to ethylene, gibberellin, and zeatin metabolism (**Figure 8**). Third, functional enrichment among upregulated genes highlights signaling and buffering processes, notably including protein phosphorylation and glutathione metabolism.

The reconstructed PPI network also contains a prominent genome surveillance and cell-cycle module (55 proteins), underscoring integrity maintenance as a coordinated component of MST MTs development (**Figure 8**). These features define MST resilience as an integrated regulatory state driven by transcriptional remodeling, hormone connectivity, and redox buffering.

Rather than broad metabolic activation, the GO enrichment highlights specific adaptive modules, including phenylpropanoid metabolism, redox regulation, and cell wall remodeling, which collectively support structural reinforcement and stress buffering during MT development under MST conditions. The enrichment of lipid and hormone-related processes further suggests coordinated membrane remodeling and signaling integration, providing a comprehensive framework for potato adaptation to multi-stress environments.

### 4.1 Developmental reprogramming under MST: the central role of the StSP6A module

**Figure 14:**
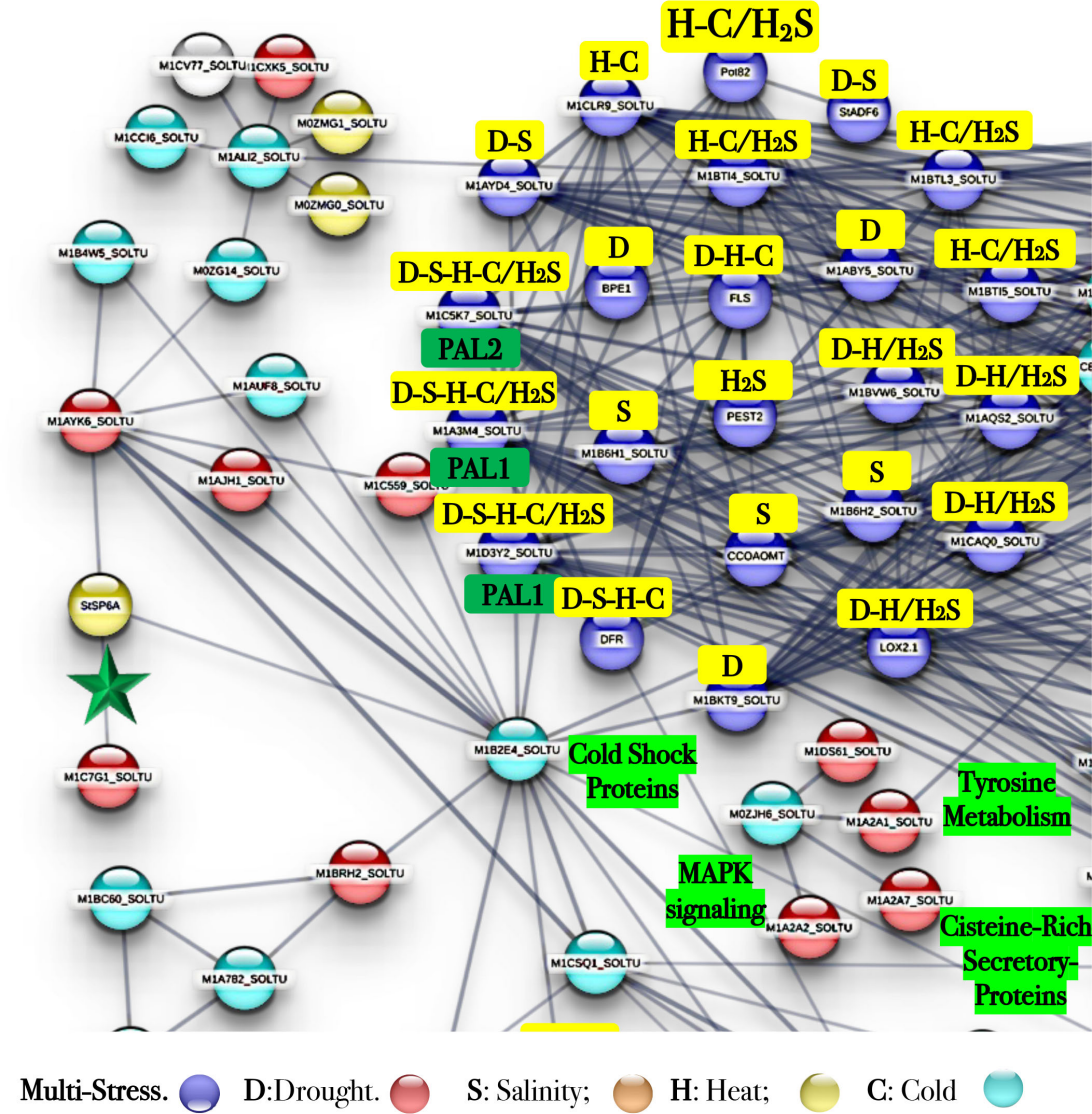
Amplification of *StSP6A* module inside PPI network of upregulated genes associated with MT development in *Solanum tuberosum* L. under MST conditions. The network was generated using the STRING database v12.0 with confidence score threshold of 0.500.

At the core of the STRING-derived interaction network, the StSP6A-FD module emerges as a highly connected hub that links tuber initiation with stress-associated metabolic and redox-related modules (**Figure 14**). StSP6A, the potato ortholog of FLOWERING LOCUS T (FT) controls tuberization competence in a dosage-dependent manner (Navarro et al., 2011; Lehretz et al., 2019).

Under a constraining environment, StSP6A overexpression increases tuber number and can enhance yield, including under ambient and heat-challenging conditions. Under combined constraints (e.g., heat with high nitrogen) or in co-engineered setting (StSP6A + HXK1), additional yield improvements have been reported (Lehretz et al., 2021).

Under elevated temperature, constitutive StSP6A overexpression restores tuber formation (tuber number) but does not fully recover yield at late stages (Park et al., 2022a), consistent with heat-induced constraint on source-to-sink sugar transport (Park et al., 2022a; Lehretz et al., 2019).

These observations indicate that StSP6A is necessary but not sufficient for productivity under heat stress, and that tuberigen control must remain coupled to carbon allocation and redox-buffering programs during MST (Park et al., 2022a).

The interaction between StSP6A and its FD-like bZIP partner (Teo et al., 2017) supports the conserved FT–FD activation module and is consistent with the tuberization transcriptional program remains functional under MST.

Importantly, the association of StSP6A with the plastid ribosomal protein RPL33 (M1AYK6), required to sustain plastid translation capacity under cold stress, link the tuberigen module to organellar translational robustness during temperature fluctuation (Rogalski et al., 2008). In potato, genome-wide RNA-seq combined with ribosome profiling shows that drought and heat substantially alter translational efficiency and can decouple translational output from mRNA abundance (Jian et al., 2023), underscoring translation as an independent constraint under MST. Consistently, under simultaneous stress (e.g., salinity, drought, and elevated temperature), quantitative proteomics in rice reveals rapid remodeling of chloroplast-associated proteins pools, in line with plastid proteostasis/translation emerging as bottleneck during complex stress exposure (Abdirad et al., 2022).

The inclusion of additional PEBP family members, including BFT-like proteins (Pin and Nilsson, 2012), and G-type lectin receptor-like kinases (Singh and Zimmerli, 2013), further supports integration of environmental inputs into FT/TFL1-like regulatory axis (Pin and Nilsson, 2012; Singh and Zimmerli, 2013). In *Arabidopsis*, *BFT (M1B4W5)* is transcriptionally induced by high salinity in an ABA-dependent manner (Ryu et al., 2014). Beyond salinity, *BFT (M1B4W5)* also responds to other abiotic cues (e.g., drought/osmotic and ABA), whereas evidence for cold effect is condition-dependent (Chung et al., 2010).

Moreover, the association of the tuberigen module with Class III peroxidase *PER7 (M1B2E4)* places tuberization within a redox-regulated framework. Class III plant peroxidases use apoplastic H_2_O_2_ to drive oxidative polymerization and covalent cross-linking in the cell wall. Alternatively, they promote wall loosening ROS-dependent wall-modifying activities, thereby coupling ROS fluxes to controlled structural remodeling (Passardi et al., 2005; Almagro et al., 2009). Specific class III peroxidases modulate cell expansion regulate cell developmental programs via RBOH-dependent production and spatiotemporal coordination (Passardi et al., 2005; Baxter et al., 2014).

Together, these connections indicate that stolon reprogramming under MST occurs within a broader redox- and environmental-sensing network. Rather than a simple developmental delay, it entails structural reorganization. This remodeling is consistent with ROS-tuned activity of Class III peroxidases together with phenylpropanoid pathway activation, a combination that safeguards signaling fidelity while reinforcing cell wall architecture during MTs development.

### 4.2 Phenylpropanoid and lignification pathways as adaptive interfaces

The enrichment of family members of the phenylpropanoid metabolism constitutes a central adaptive module under MST (Kriegshauser et al., 2021; Bhardwaj et al., 2013; Park et al., 2024; Rai et al., 2021; Cong et al., 2025; Ouyang et al., 2023). These pathways do not solely reinforce cell walls but also regulate ROS balance, UV protection, and hormonal crosstalk, positioning phenylpropanoid outputs as a flexible interface between protection and development during MTs development.

Key MST genes include *PAL1* (*M1A3M4, M1D3Y2*) and *PAL2* (*M1C5K7*), involved in phenylpropanoid biosynthesis and H_2_S signaling; *DFR* (*Q6WG04*), a key enzyme in anthocyanin biosynthesis, *OMT1* (*M1CLR9)* methylates flavonoids and monolignols; and the glycosyltransferases *UGT72D1* (*M0ZZL3*) and *UGT72E1* (*M1AYD4*) stabilize monolignols.

This module also contains the cytoskeletal regulators *Actin-12* (*ACT12* (*Pot82*)) and *ADF6 (StADF6)*, consistent with maintenance of structural integrity under stress and potential linkage to H_2_S signaling, as well as *LOX1* (*M1AQS2, M1CAQ0, M1BVW6*) and *LOX2 (LOX2.1)*, which catalyze oxylipin production and are connected to H_2_S-associated responses.

The cell wall remodeling components such as *PME3* (*M1AIV9*) and pectin lyase-like proteins, further support stress-tuned wall plasticity during MTs initiation. In addition, *STR18* (*M0ZVS2*) a rhodanese domain-containing thiosulfate sulfurtransferase, reinforces a linkage between phenylpropanoid/wall outputs and sulfur-redox chemistry, while *PLDDELTA* (*M1ALP3*) points to concurrent membrane remodeling and lipid signaling.

Consistent with the MST structure of the network, phenylpropanoid and wall-associated components may point to context-dependent response patterns rather than a single reinforcement program. Under drought-salinity-cold, flavonol-related routes (e.g., *FLS1 (FLS)*) are consistent with enhanced antioxidant buffering (Han et al., 2023; Ma et al., 2024). Under salinity-cold, pectin lyase families support wall remodeling linked to freezing tolerance (Gall et al., 2015). Under drought-salinity contexts, genes such as *CCOAOMT1 (CCOAOMT)* are associated with wall reinforcement, while *ANNAT8* (*M1ABY5*) supports membrane stability (Konopka-Postupolska et al., 2009; Giordano et al., 2016; Niu et al., 2025). Under drought–heat, *PME18* (*BPE1*) aligns with adjusted pectin methylesterification and wall plasticity (Phan et al., 2007). These patterns are consistent with phenylpropanoid-associated pathways function not only in structural reinforcement, but also as a modular interface for tuning adaptive responses during MTs development.

Importantly, this aligns with our earlier structural observation that potato exhibits an expanded repertoire of stress-responsive TFs (MYB, WRKY, ERF) compared to *Arabidopsis*. In species such as *A. thaliana*, lignification is largely controlled by a development-centered NAC–MYB hierarchy. In contrast, potato may integrate stress-inducible transcriptional layers into this module, suggesting evolutionary diversification toward environmental plasticity. Accordingly, lignification in potato may function as a dynamic interface between structural development and stress acclimation, particularly during the vulnerable MTs stage.

### 4.3 Hydrogen sulfide, as a crosstalk hub in MST integration

Hydrogen sulfide (H_2_S) has been present since early life on Earth and was later integrated into mitochondrial respiration during plant evolution. It activates cyanoamino acid metabolism, fatty acid degradation, endoplasmic reticulum-associated protein processing, and ribosome biogenesis.

**Figure 15:**
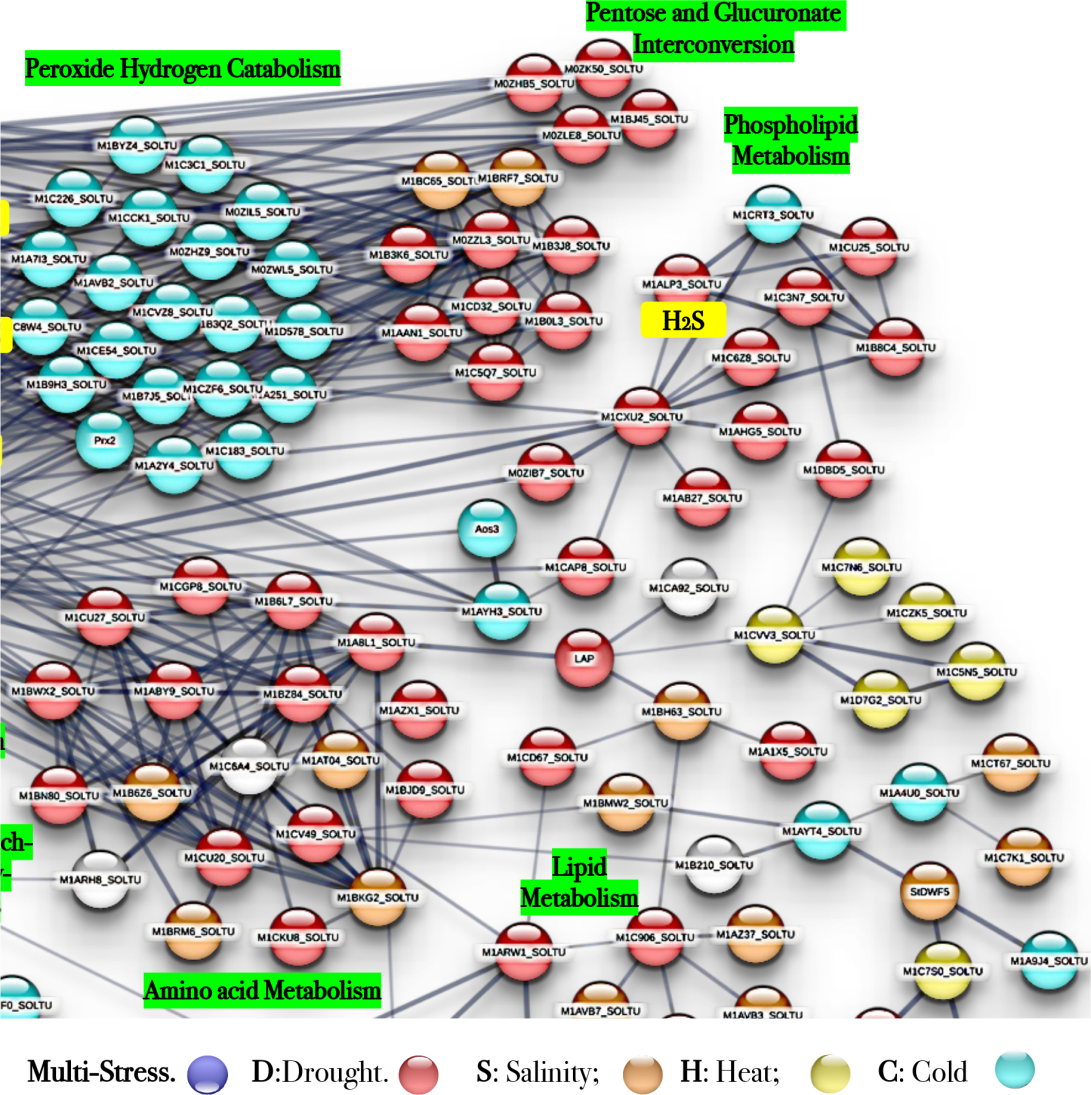
Amplification of *H_2_S* module inside PPI network of upregulated genes associated with MT development in *Solanum tuberosum* L. under MST conditions. The network was generated using the STRING database v12.0 with confidence score threshold of 0.500.

It acts as a central hub in second messenger signaling, including Ca^2+^, methylglyoxal, ABA, and H_2_O_2_. Additionally, H_2_S increases photosynthetic activity by increasing grana lamellae and carboxylation efficiency via Rubisco activity. In *A. thaliana*, 189 H_2_S-associated genes connect MAPK cascades, hormone signaling, phenylpropanoid biosynthesis, and transcriptional regulation linked to Ca^2+^-associated process (Olson and Straub, 2016; Zhao et al., 2020) (**Figure 15**).

Our transcriptomic and phenotypic data suggest that hydrogen sulfide (H_2_S) signaling may act as a key integrative component during potato microtuberization under MST. We observed a consistent upregulation of genes associated with sulfur metabolism and H_2_S-related pathways, such as *STR18*, *PAL1*, *OMT1*, *LOX1*, *PER7*, and *PLDDELTA*.

These results, further validated by qPCR, functionally link H_2_S signaling to phenylpropanoid metabolism, redox homeostasis and cell wall remodeling in potato.

While H_2_S has been demonstrated to induce tolerance against various adverse environmental conditions, such as drought, salinity, high temperature, cold, and heavy metal stress by promoting the accumulation of osmolytes (proline, betaine and trehalose) and modulating antioxidant pools, supplying sulfur to cells (Pandey and Gautam, 2020), our findings place these pathways within the MT development under MST conditions.

In our reconstructed PPI network, H_2_S-associated enzymes were distributed across multiple modules, particularly those related to phenylpropanoid metabolism, redox buffering, and membrane remodeling. This supports the hypothesis that H_2_S signaling is embedded within a broader regulatory framework coordinating metabolic and structural adaptation under combined stress.

A significant finding in the MST dataset was the induction of TFs such as bZIP, NAC, WRKY, and MYB, which are present in H_2_S-related gene promoter sequences. In *Arabidopsis*, H2S-mediated upregulation of drought-responsive genes such as *DREB2A*, *DREB2B*, *RD29A* and *CBF4*, has been observed, and interacts with ABA-dependent signaling to induce stomatal closure and reduce water loss within guard cells (Pandey and Gautam, 2020).

The recurrence of H_2_S-related enzymes in potato suggests a conserved, and potentially prominent, role in MST adaptation. H_2_S may therefore operate at multiple levels, including redox buffering, modulation of WRKY/MYB/bZIP TFs, coordination of phenylpropanoid metabolism, and Ca^2+^-linked signaling and within ABA- and ethylene-associated pathways.

Despite the strong correlative evidence provided by the induction of genes like *STR18* and the response to NaHS, we acknowledge certain limitations. Endogenous H_2_S levels were not directly quantified in the potato MTs, and the specific regulatory function of the identified genes requires further validation. Therefore, while our data establish a mechanistic framework linking sulfur metabolism to MST resilience in *S. tuberosum*, the proposed role of H_2_S should be interpreted as a hypothesis supported by correlative evidence rather than direct causation.

Future research should include more detailed dose-response curves and direct gasotransmitter measurements to fully decipher the H_2_S-mediated signaling during potato tuberization.

### 4.4 Hierarchical transcriptional architecture: insights from the PCA analysis

The PCA analysis revealed that MYB and bHLH/MYC families dominate global transcriptional variance (Dim.1), while HSF and WRKY contribute mainly to secondary differentiation (Dim.2). This pattern supports a hierarchical regulatory model, in which primary integrators (MYB, bHLH/MYC) coordinate broad metabolic and structural reprogramming, while secondary the modulators (HSF, WRKY) fine-tune stress-specific outputs.

This architecture is consistent with the expanded TFs repertoire identified in potato and suggests that MST adaptation relies on a limited number of transcriptional hubs that control extensive downstream networks.

### 4.5 Metabolic reallocation: carbohydrate, lipid, and membrane remodeling

**Figure 16:**
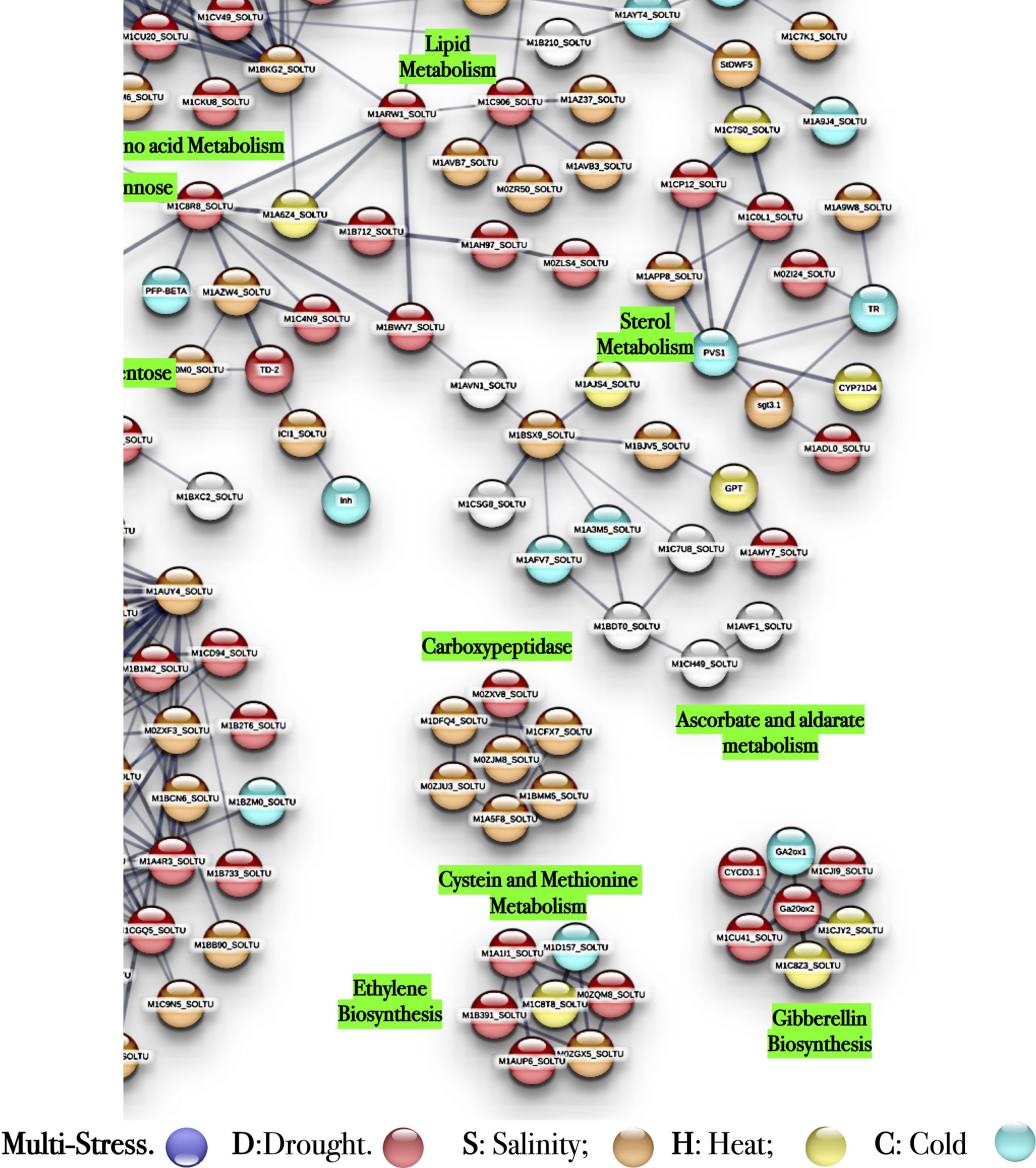
Amplification of metabolic reallocation module inside PPI network of upregulated genes associated with MT development in *Solanum tuberosum* L. under MST conditions. The network was generated using the STRING database v12.0 with confidence score threshold of 0.500.

MST adaptation during MTs development also requires metabolic redistribution. *SUS4 (M0ZT40)* and *CWINV1 (M0ZRR7)* support sucrose partitioning toward starch biosynthesis and sugar-mediated signaling (Sonnewald et al., 1997; Tauberger et al., 1999), while *HKT1 (M1CXU4)* contributes to ionic homeostasis under salinity (Ali et al., 2019) (**Figure 16**).

β-glucosidases (e.g., *BGLU17 (M1A782)* and *BGLU42 (M1BC60)*) may modulate conjugated metabolite pools, linking carbohydrate turnover with stress-response signaling. However, the most direct functional evidence is for *BGLU42 (M1BC60)*, which acts downstream of MYB72-associated regulatory programs and has been shown to hydrolyze scopolin and other β-glucosides (Zamioudis et al., 2014; Horikoshi et al., 2022).

Lipid remodeling enzymes (PLDDELTA (M1ALP3), PLA2-ALPHA (M1CXU2), NPC6 (M1B8C4)) contribute to membrane dynamics and lipid-derived signaling, helping stabilize compartments during osmotic and temperature fluctuations (Distéfano et al., 2015; Romero et al., 2014). Sterol and terpenoid biosynthetic genes (e.g., *CAS1 (M1B210), SMO1-1 (M1AYT4), DWF5 (M1A5L3), MVD2 (M1C7S0), GGPPS1/GGR (M1CP12; M1C0L1)*, and *TPS21 (M1APP8)*) further support membrane stability and the formation of stress-responsive signaling platforms (Zhan et al., 2022).

In the MST PPI network, several proteins were identified as non-specific lipid transfer proteins (LTPs), including LTP3 (M1AVB7) and LTP12 (M1AVB3) as well as LTP1 (M0ZR50 and M1AZ37). These proteins bind and transport lipids molecules required for membrane dynamics and cuticle assembly during stress responses. Consistently, LTPs participate in plant responses to abiotic stresses such as drought, salinity, osmotic stress, freezing, and extreme temperatures (Liu et al., 2015).

In agreement with previous integrative multi-omics analysis of combined stress responses (drought and heat) in plants (Priya et al., 2023), which highlight the enrichment of amino acid, energy, and carbohydrate metabolism pathways, our transcriptomic data similarly revealed significant enrichment of amino acid metabolism and carbohydrate metabolism pathways. Notably, cysteine metabolism, glycolysis, and the metabolism of fructose, mannose, ascorbate, aldarate, and sugar metabolism were prominently represented, supporting the idea that central metabolic reprogramming is a common feature of plant responses to complex stress conditions.

### 4.6 Physiological support and redox homeostasis

V-ATPase subunits maintain vacuolar pH, ensuring ionic and osmotic balance (Schumacher and Krebs, 2010). Carbonic anhydrases buffer CO_2_/HCO_3_^-^ ratios, protecting photosynthetic and metabolic processes under stress (Polishchuk, 2021; DiMario et al., 2017). UGTs glycosylate metabolites, contributing to metabolic stabilization and detoxification during MST conditions (Lim and Bowles, 2004; Caputi et al., 2012).

These findings suggest that MTs development under MST involves not only transcriptional regulation but also coordinated restructuring of carbon fluxes and membrane composition, enabling sustained developmental progression despite environmental constraints.

### 4.7 Genome stability and cell cycle control under MST

**Figure 17:**
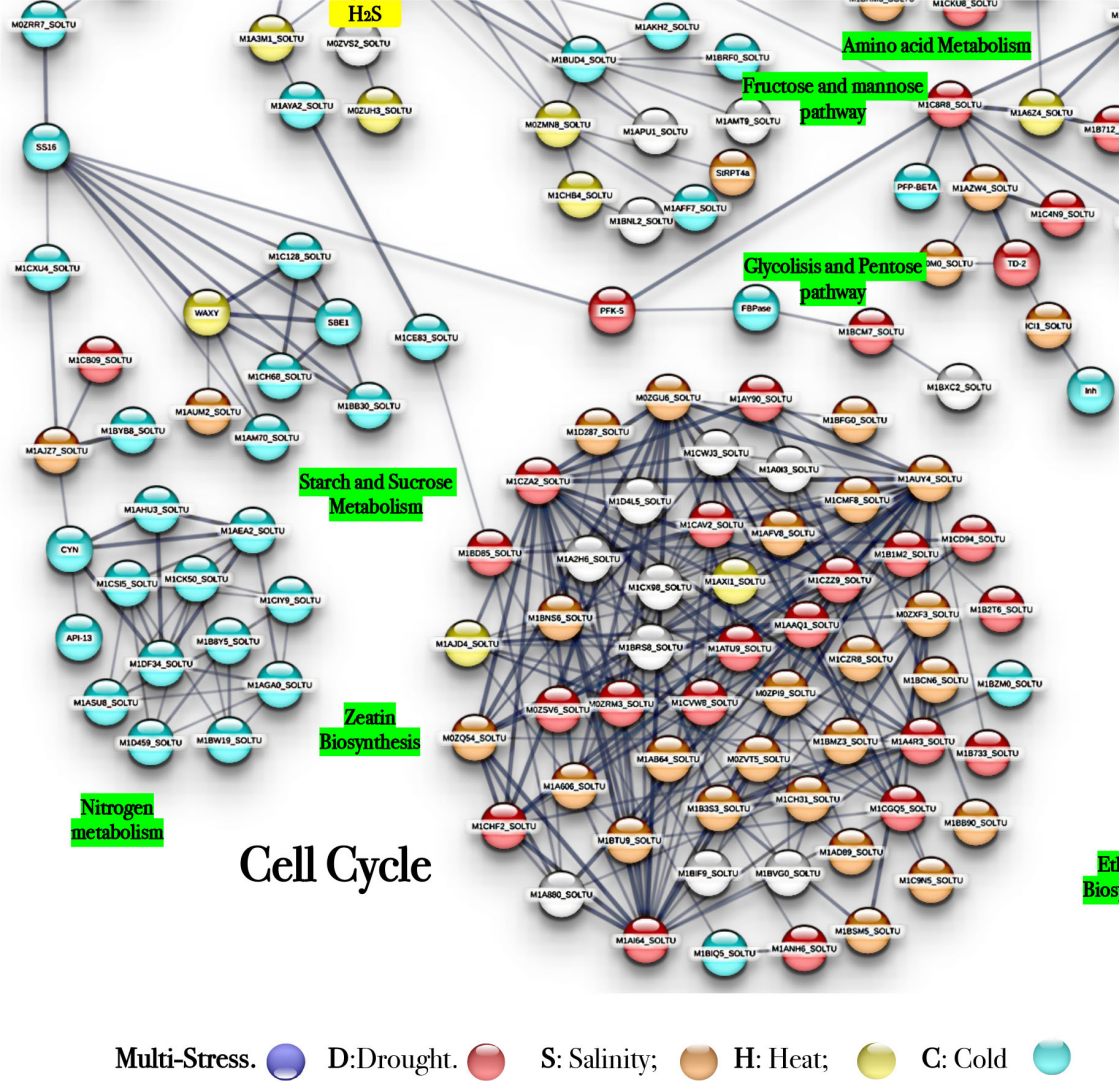
Amplification of cell cycle control module inside PPI network of upregulated genes associated with MT development in *Solanum tuberosum* L. under MST conditions. The network was generated using the STRING database v12.0 with confidence score threshold of 0.500.

Potato maintains cell-cycle progression under oxidative and osmotic stresses through activation of D-type cyclins, replication factors, condensins, and DNA repair proteins (Morgan, 2007; Masai et al., 2010) (**Figure 17**). Rather than halting growth, mitotic activity is balanced with genome surveillance, suggesting that stress tolerance during MTs development depends on safeguarding genome integrity while sustaining controlled cell division.

Cell-cycle checkpoints (G1/S and G2/M) act as central hubs integrating proliferation with environmental adaptation (Qi and Zhang, 2020). Abiotic stresses can delay mitotic entry and activate DNA damage control pathways (Kitsios and Doonan, 2011). Consistently, our protein–protein interaction network revealed a cell-cycle module of 55 proteins, including 11 G2/M checkpoint components and 12 DNA damage prevention/recovery factors, highlighting the centrality of genome surveillance under MST.

Key regulators of replication and S-phase include PHL11 (M1AJD4), CDC6 (M1CZA2), ORC4/5 (M1CZZ9/M0ZGU6), and the MCM helicase complex (MCM2 (M0ZQ54), MCM5 (M1AUY4), MCM7 (M1AI64)), which link replication competence to heat and cold-associated genome protection (Bell and Dutta, 2002; Ni et al., 2009; Xi et al., 2020; Rankin and Rankin, 2024).

Cyclin-CDK complexes (CYCA2-4 (M1CGQ5, M0ZRM3), CYCA3-2 (M1ATU9), CYCA1-1 (M1A606), CYCB1-2 (M1AAQ1), SDS (M1CVW8)) orchestrate G1/S and G2/M transitions, responding to ROS and microtubule disruption under heat stress (Kitsios and Doonan, 2011). Condensin subunits, F19K16.15 (M1BD85) RAD3-like helicases, kinesins, and MAP65-3 (M1D287) maintain mitotic architecture and spindle integrity, supporting stable chromosome segregation during stress (Schubert et al., 2013; Gao et al., 2017; Zhang et al., 2022; Van Dieren et al., 2026).

Cell-cycle regulation is further integrated with redox and developmental buffering, as DUT (M1A2H6, M1CAV2) interacts with glyoxal oxidase to generate extracellular H_2_O_2_ for peroxidase-mediated lignin processes, while IAA29 (M1B2T6) contributes to genome integrity and antioxidant programs during tuberization (Liu et al., 2024; Wang et al., 2018; Takahashi et al., 2025).

### 4.8 Integrative model of MST adaptation in potato

The simultaneous exposure to drought, salinity, heat, and cold (D-S-H-C) induces a regulatory state in which MTs development is sustained through the integrated action of developmental, metabolic, redox, structural, and genome maintenance processes.

Potato stolon explants under MST conditions activate StSP6A–FD complex, reprogramming ancient mechanisms of abiotic stress adaptation. In this context, the biopolymer barriers composed of lignin, cutin, suberin are reinforced through the action of AP2/ERF, MYB, bHLH/MYC transcription factors, coupled with the versatile multi-stress regulator hydrogen sulfide (H_2_S) signaling. The coordinated activity of enzymes including PAL1 (M1A3M4, M1D3Y2)/PAL2 (M1C5K7), OMT1 (M1CLR9), LOX1 (M1AQS2), PER7 (M1B2E4), laccases, and pectinesterases, together with proteins associated with pectin dynamics and lipid metabolism, supports the establishment of a cellular matrix with mechanical and redox properties suitable for MTs development under multi-stress.

The recurrence of STR18 (M0ZVS2), PALs, LOX1 (M1AQS2), PLD (M1ALP3), and OMT1 (M1CLR9) in response to H_2_S signaling suggests that this gasotransmitter modulates tissue antioxidant capacity, adjusts peroxidase activity, and contributes to cell wall polymer deposition. Its interaction with pathways associated with ABA, ethylene, and gibberellins indicates that this axis integrates redox homeostasis and hormonal regulation, thereby promoting the persistence of a physiological state conducive to tuberization during D-S-H-C exposure.

In this context, the H_2_S-redox regulatory axis functions as a key intersection, linking oxidative adjustments with cell wall dynamics and primary metabolism, while modulating ROS availability, peroxidase activity, and structural polymer formation.

Maintenance of genome integrity is supported by D-type cyclins, replication factors, condensins, and DNA repair proteins that regulate cell cycle progression and cell division.

The enrichment of amino acid and carbohydrate metabolic pathways, including cysteine metabolism, glycolysis, and sugar metabolism routes, highlights central metabolic reprogramming as a core component of plant adaptive responses to drought and heat stresses.

**Figure 18:**
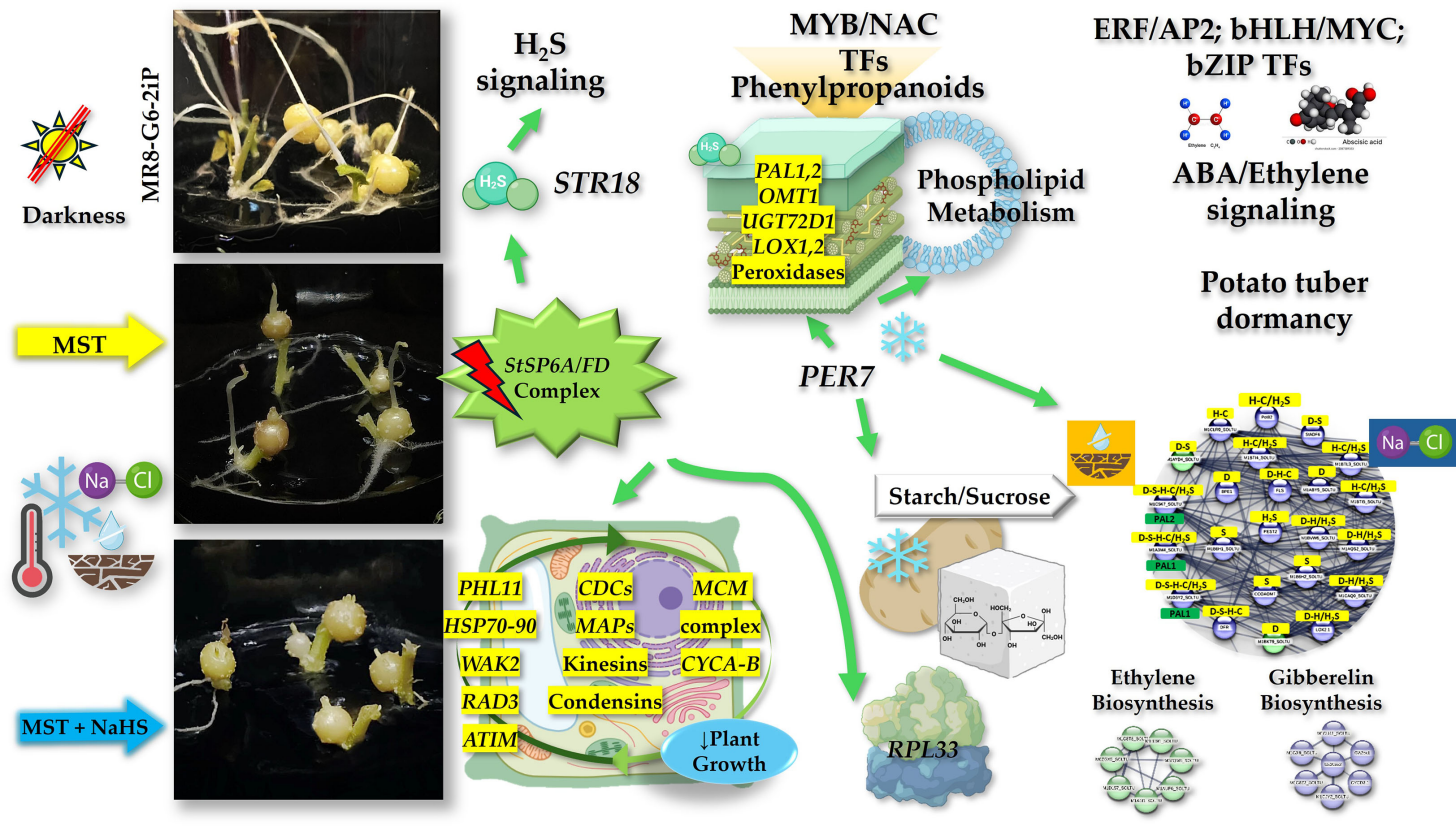
Proposed model of the transcriptomic analysis of MTs development under MST conditions of *potato S. tuberosum* L. cv. Alpha.

Thus, targeted editing of central regulators of sulfur metabolism and lignin biosynthesis within this network may enhance MST resilience in potato cultivars through chromatin remodeling under increasingly severe climate change scenarios (**Figure 18**).

Collectively, our data support a model in which potato integrates developmental, metabolic, and stress-signaling modules into a highly interconnected regulatory framework.

## 5 Conclusions

Microtuber (MT) development under sequential multi-stress treatments (osmotic stress, 50 mM NaCl, 38 °C for 24 h, followed by 4 °C for 24 h) activates 1,475 genes organized into a protein–protein interaction network of 317 components across 17 functional modules, reflecting a regulatory state specific to combined stress rather than a simple additive response to single stressors.

MT development under MST is associated with the upregulation of genes involved in lignin and suberin biosynthesis, suggesting the activation of protective barrier biopolymers regulated by MYB transcription factors and phenylpropanoid enzymes. Wax and cutin biosynthesis, along with cell-wall remodeling and redox-related processes, also involve antimicrobial peptides and key enzymes such as pectinesterases, pectin lyases, and laccases.

Eleven proteins linked to hydrogen sulfide (H_2_S) signaling, including OMT1, LAP2, PAL1, PAL2, lipoxygenases, phospholipase D, and rhodanese, were identified within the MST network, highlighting the role of H_2_S in coordinating barrier formation.

Concurrently, metabolic reprogramming of amino acid and carbohydrate pathways, including cysteine metabolism and glycolysis, underpins these adaptive responses. Genome integrity is maintained via the activation of D-type cyclins, replication factors, condensins, and DNA repair proteins, while 96 transcription factors, dominated by MYB and bHLH/MYC families, regulate global transcriptional variance.

Overall, our findings establish a comprehensive regulatory framework that integrates microtuber induction with metabolic, redox, and transcriptional networks, unveiling the molecular basis of multi-stress resilience in potato and providing a blueprint for enhancing crop adaptation to complex environmental challenges. To build upon these findings, we propose future studies incorporating single-stress and factorial designs to resolve the individual contributions and the complex interplay of each stress factor.

## Data Availability

The dataset presented in this study can be found in NCBI BioProject online repository with the accession number PRJNA1456097, and can be found below: https://www.ncbi.nlm.nih.gov/bioproject/1456097.
