# Supplementary material for: Molecular resilience: genetic analysis of multiple-stress tolerance (osmotic, salinity, cold and heat) during potato (Solanum tuberosum L.) microtuberization
Source: Front Plant Sci. 2026 May 29;17:1828205. doi: 10.3389/fpls.2026.1828205 (PMC13261755; doi:10.3389/fpls.2026.1828205)
Supplement: Supplementary file 1 [file Table1.docx]

**Supplementary Material.**

**Validation of transcriptome data by qPCR in MST Response**

qRT-PCR reactions were performed under the following conditions: initial denaturation at 95°C for 10 min, followed by 40 cycles of 95°C for 15 s, annealing at 60°C for 60 s, and extension at 72 °C for 30 s. A melt curve analysis was included to verify amplification specificity.

**Endogenous genes**

| **String v12.0** | **NCBI ID** | **OLIGO NAME** | **SEQUENCE** |
| --- | --- | --- | --- |
| SEC3(M1B909) | XM_006342542.2 | SEC3 For | GATCTGCGGAAGGTGGTAAA |
|  |  | SEC3 Rev | CAGCAACTCCTCTGAGGTTAAG |
| Ef1-alpha(Ef-p) | NM_001288491.1 | Ef1-alpha For | GCACTGGAGCATATCCGTTT |
|  |  | Ef1-alpha Rev | TTTGGCCCTACTGGTTTGAC |

**Selected genes**

| **String v12.0** | **NCBI ID** | **OLIGO NAME** | **SEQUENCE** |
| --- | --- | --- | --- |
| FT(StSP6A) | NM_001287968.1 | StSP6A For | CCCTGACTTGAGGGAGTATCTA |
|  |  | StSP6A Rev | GTAGGTGTTGGATTCTCGTAGC |
| PER7(M1B2E4) | XM_006364783.2 | PER7 For | GCACAGTTTCCAACGCTAAAG |
|  |  | PER7 Rev | GGACAACCAAGTCGAGAACA |
| PAL1(M1D3Y2) | XM_006367472.2 | PAL1 For | GGAGCTTTCGAGGACGAATTA |
|  |  | PAL1 Rev | GATCCTGTTAGGAATCGAAGGG |
| LOX1(M1AQS2) | NM_001287987.1 | LOX1 For | CGCCTCAAGAACTTTGCTATTC |
|  |  | LOX1 Rev | ACCAAGTTCATCGCCATCT |
| PHL11(M1AJD4) | XM_006352877.2 | PHL11 For | GAAGAGCCTGTCCACTGATATG |
|  |  | PHL11 Rev | GTCCGTGCATGTAGTCCATTA |
| OMT1(M1CLR9) | XM_006350136.2 | OMT1 For | GAGGCCATTCTACCAGTGAAA |
|  |  | OMT1 Rev | CGAGGGATCGAAATTTGTGTTG |
| STR18(M0ZVS2) | XM_006340685.2 | STR18 For | GTGAGGTCCCTCTATGCTACTA |
|  |  | STR18 Rev | CTGCTGCAATCAAAGCTCATC |
| LAC12(M1C7N6) | XM_006353356.2 | LAC For | AGCACATCACCAGGGAATAC |
|  |  | LAC Rev | CCAGAGTGTCCTGCTAACATT |
| LAP2(LAP) | NM_001318637.1 | LAP For | TGCTCTTGGACCTTCAGTTG |
|  |  | LAP Rev | TCCCAGTAACTCTCCTCCATAG |
| LTP1(M0ZR50) | XM_006345207.2 | LTP1 For | GAGGGTGTTGTGGTGGTATT |
|  |  | LTP1 Rev | TAGCACTAGCGGCTGATTTG |
